# Multiobjective triclustering of time-series transcriptome data reveals key genes of biological processes

**DOI:** 10.1186/s12859-015-0635-8

**Published:** 2015-06-26

**Authors:** Anirban Bhar, Martin Haubrock, Anirban Mukhopadhyay, Edgar Wingender

**Affiliations:** 1 Institute of Bioinformatics, University Medical Center, Georg August University, Goettingen, Goldschmidtstrasse 1, Goettingen, D-37077 Germany; 2 Department of Computer Science and Engineering, University of Kalyani, Kalyani, -741235 India

**Keywords:** Microarray gene expression data, Developmental biology, Tricluster, Multi-objective optimization, Eigen gene, Affirmation score, TRANSFAC

## Abstract

**Background:**

Exploratory analysis of multi-dimensional high-throughput datasets, such as microarray gene expression time series, may be instrumental in understanding the genetic programs underlying numerous biological processes. In such datasets, variations in the gene expression profiles are usually observed across replicates and time points. Thus mining the temporal expression patterns in such multi-dimensional datasets may not only provide insights into the key biological processes governing organs to grow and develop but also facilitate the understanding of the underlying complex gene regulatory circuits.

**Results:**

In this work we have developed an evolutionary multi-objective optimization for our previously introduced triclustering algorithm *δ*-TRIMAX. Its aim is to make optimal use of *δ*-TRIMAX in extracting groups of co-expressed genes from time series gene expression data, or from any 3D gene expression dataset, by adding the powerful capabilities of an evolutionary algorithm to retrieve overlapping triclusters. We have compared the performance of our newly developed algorithm, EMOA- *δ*-TRIMAX, with that of other existing triclustering approaches using four artificial dataset and three real-life datasets. Moreover, we have analyzed the results of our algorithm on one of these real-life datasets monitoring the differentiation of human induced pluripotent stem cells (hiPSC) into mature cardiomyocytes. For each group of co-expressed genes belonging to one tricluster, we identified key genes by computing their membership values within the tricluster. It turned out that to a very high percentage, these key genes were significantly enriched in Gene Ontology categories or KEGG pathways that fitted very well to the biological context of cardiomyocytes differentiation.

**Conclusions:**

EMOA- *δ*-TRIMAX has proven instrumental in identifying groups of genes in transcriptomic data sets that represent the functional categories constituting the biological process under study. The executable file can be found at http://www.bioinf.med.uni-goettingen.de/fileadmin/download/EMOA-delta-TRIMAX.tar.gz.

**Electronic supplementary material:**

The online version of this article (doi:10.1186/s12859-015-0635-8) contains supplementary material, which is available to authorized users.

## Background

One of the main aims of functional genomics is to understand the dynamic features encoded in the genome such as the regulation of gene activities. It often refers to high-throughput approaches devised to gain a complete picture about all genes of an organism in one experiment. Several steps, such as transcription, RNA splicing and translation are involved in the process of gene expression, which is subject to a great many of regulatory mechanisms. Analysis of such gene expression data provides enormous leverages to understand the principles of cellular systems, diseases mechanisms, molecular networks etc. Genes having similar expression profiles are frequently found to be regulated by similar mechanisms. Previous studies elucidated the impact of highly connected intra-modular hub genes on such regulations [1–3]. Detecting hub genes and analyzing their roles may facilitate understanding the basal control mechanisms of a certain normal or disease cellular phenotype to develop.

Microarray technology is used to measure the expression of thousands of genes over a set of biological replicates simultaneously. In recent years, such expression signatures have increasingly been monitored for sets of time points in order to follow the course of biological processes. In case of such three-dimensional datasets, at each time point the activity of all genes is measured for a number of biological replicates. Although the experimental setups are kept identical for these replicates, variations between them can still occur. For instance, stochastic effects can result in delays or accelerations of a certain cell state transition. Thus, grouping similar biological replicates may facilitate the analysis of time series gene expression data. Moreover, expression profiles of genes may also vary over different time points. Appropriate computational methods are therefore required to analyze such high-throughput datasets specifically to identify temporal expression patterns over biological replicates and time points. Clustering, one of the unsupervised learning approaches, has been used to explore such two-dimensional gene expression datasets. Clustering algorithms aim to maximize similarity within or to minimize similarity between clusters, based on a distance measure [4]. Clustering is able to group genes or samples over a set of samples or genes, respectively, but it has been reported in previous studies that genes are not necessarily to be co-expressed over all samples. Hence to find such local patterns, i.e. genes having similar expression profiles over a subset of samples in 2D gene expression datasets biclustering algorithms are used [5]. In previous studies, biclusters have been found to be biologically more significant as these algorithms aim to extract groups of correlated genes from a subset of samples. Such subspace clustering techniques find clusters in multiple overlapping subspaces. To deal with time series gene expression datasets, biclustering algorithms fail to extract genes that have similar expression profiles over a subset of samples during a subset of time points. To perform co-expression analysis in such three-dimensional gene expression datasets triclustering algorithms have to be employed. Zhao et al. proposed the TRICLUSTER algorithm that aims to retrieve groups of genes that have similar expression profiles over a subset of samples during a subset of time points [6]. In a recent work, Tchagang et al. proposed a triclustering algorithm (OPTricluster) for mining short time series gene expression datasets. OPTricluster effectively mines time series gene expression data having approximately 3-8 time points and 2-5 samples. According to their definition of a tricluster, genes belonging to a tricluster must have constant, coherent or order preserving expression patterns over a subset of samples during a subset of time points. In case of an order-preserving tricluster, there must be a permutation of the time points such that expression levels of genes form a monotonic function [7]. In our previous work we have proposed a triclustering algorithm *δ*-TRIMAX by introducing a novel mean squared residue score (MSR) to mine a 3D gene expression dataset and each tricluster must have an MSR score below a threshold *δ* [8, 9]. In spite of its proven merits [8, 9], *δ*-TRIMAX has some limitations: a) it can not retrieve overlapping triclusters, b) due to its greedy approach it often gets stuck at local optima. Finding overlapping triclusters is important in biological context, since each gene may participate in several biological processes, thus being subject to multiple regulatory influences [10]. A subset of genes may therefore be involved in a set of biological processes and consequently belong to several triclusters. However, the goals of *δ*-TRIMAX algorithm were to maximize the volume and minimize the MSR score of the resultant triclusters. Hence the problem of optimizing such multiple conflicting objectives can be classified as multi-objective optimization problem where a set of alternative solutions of equivalent quality exists instead of one single optimal solution. To optimize the conflicting objectives of *δ*-TRIMAX we have used a non-dominated sorting genetic algorithm-II (NSGA-II) [11] as a multi-objective optimization method to develop EMOA- *δ*-TRIMAX (Evolutionary Multi-objective Optimization Algorithm for *δ*-TRIMAX). It could demonstrate that EMOA- *δ*-TRIMAX effectively copes with the problems of *δ*-TRIMAX.

**Fig. 1:**
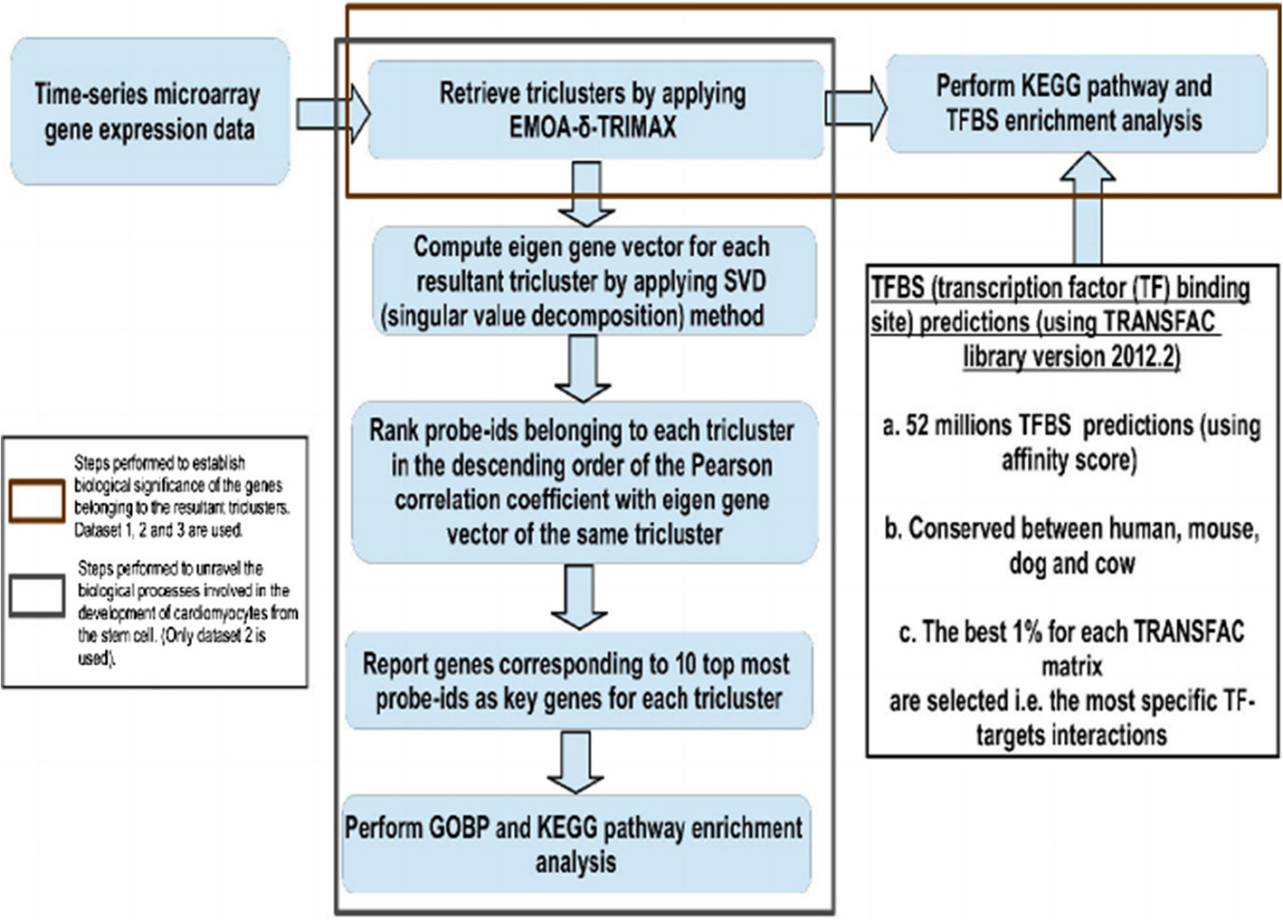
Workflow. General workflow applied in this work

The main purpose of studying developmental biology is to gain insight into the biological processes by which an organism, or one particular organ, grows and develops. Cell differentiation refers to the biological processes by which a less specialized cell develops into a specialized cell type. For instance, stem cells can differentiate into different specialized cell types such as cardiomyocytes, neural progenitors etc. [12, 13]. In this work we aim at analyzing gene expression profiles during the differentiation of human induced pluripotent stem cells (hiPSCs) into cardiomyocytes in order to reveal key genes, potential biological processes and/ or pathways by which stem cells gain new phenotypic features of adult heart cells. To study the temporal expression patterns over developmental time points and biological replicates, we have applied our proposed triclustering algorithm EMOA- *δ*-TRIMAX on a real-life dataset that contains mRNA expression profiles of hiPSCs differentiating into cardiomyocytes [12]. Figure 1 shows the general work flow of this work. After retrieval of triclusters by applying EMOA- *δ*-TRIMAX we first performed enrichment analyses of KEGG pathways and transcription factor binding sites (TFBSs) among the clustered genes to demonstrate biological significance of each resultant tricluster. In the next step, we identified key genes for each resultant tricluster and performed biological process and KEGG pathway enrichment analysis to uncover potential biological processes that may govern stem cell differentiation towards adult heart.

## Methods

### Definitions

Time series gene expression dataset (D): Such a dataset can be modeled as a G × C × T matrix, of which each element *d*_*ijk*_ corresponds to the expression value of the *i*th gene over the *j*th sample and across the *k*th time point where i ∈ (*g*_1_,*g*_2_,....,*g*_*G*_), j ∈ (*c*_1_,*c*_2_,....,*c*_*C*_), k ∈ (*t*_1_,*t*_2_,....,*t*_*T*_).

Tricluster (M): A tricluster can be defined as a sub-matrix M(I,J,K) = [ *m*_*ijk*_], where i ∈ I, j ∈ J, k ∈ K. Sub-matrix M represents a subset of genes (I) that have similar expression profiles over a subset of samples (J) during a subset of time points (K).

Perfect shifting tricluster: A tricluster M(I,J,K) is called perfect shifting tricluster if each element of the tricluster is represented as: *m*_*ijk*_=*Γ*+*α*_*i*_+*β*_*j*_+*η*_*k*_, where *Γ* is a constant value of the tricluster and *α*_*i*_, *β*_*j*_ and *η*_*k*_ are the shifting factors of *i*th gene, *j*th sample, *k*th time point respectively.

Mean squared residue: Mean squared residue score (MSR) of shifting tricluster M(I,J,K) can be modeled as [8, 9]

(1) $$ \begin{aligned} MSR =&\, \frac{1}{|I||J||K|} \sum_{i \in I, j \in J, k \in K} r^{2}_{ijk} = \frac{1}{|I||J||K|} \sum_{i \in I, j \in J, k \in K}\\ &(m_{ijk} - m_{iJK} - m_{IjK} - m_{IJk} + 2m_{IJK})^{2}, \end{aligned}  $$

where the mean of the *i*th gene is $ m_{\textit {iJK}} = \frac {1}{|J||K|}\sum _{j \in J, k \in K} m_{\textit {ijk}}$, the mean of the *j*th sample is $m_{\textit {IjK}} = \frac {1}{|I||K|} \sum _{i \in I, k \in K} m_{\textit {ijk}}$, the mean of the *k*th time point is $m_{\textit {IJk}} = \frac {1}{|I||J|} \sum _{i \in I, j \in J} m_{\textit {ijk}}$, and the mean of tricluster is $m_{\textit {IJK}} = \frac {1}{|I||J||K|} \sum _{i \in I, j \in J, k \in K} m_{\textit {ijk}}$.

The MSR score of a tricluster represents the level of coherence among the elements of the tricluster. Hence a lower MSR score means better quality of a tricluster. For a perfect shifting tricluster the MSR score is zero. If we use some global normalization, like min–max normalization globally on the whole dataset, it does not affect the algorithm. Moreover, it can be shown that gene–wise Z-normalization only on a tricluster does not affect the MSR score. However, when we apply similar normalization on the whole dataset, it affects the triclusters, and in turn affects our algorithm. Still we prefer to normalize the dataset in order to eliminate the variability in gene expression profiles due to experimental errors and noises and as normalization reduces the effects of scaling patterns, scaling patterns could also be identified partially.

### Steps of EMOA- *δ*-TRIMAX

**Fig. 2:**
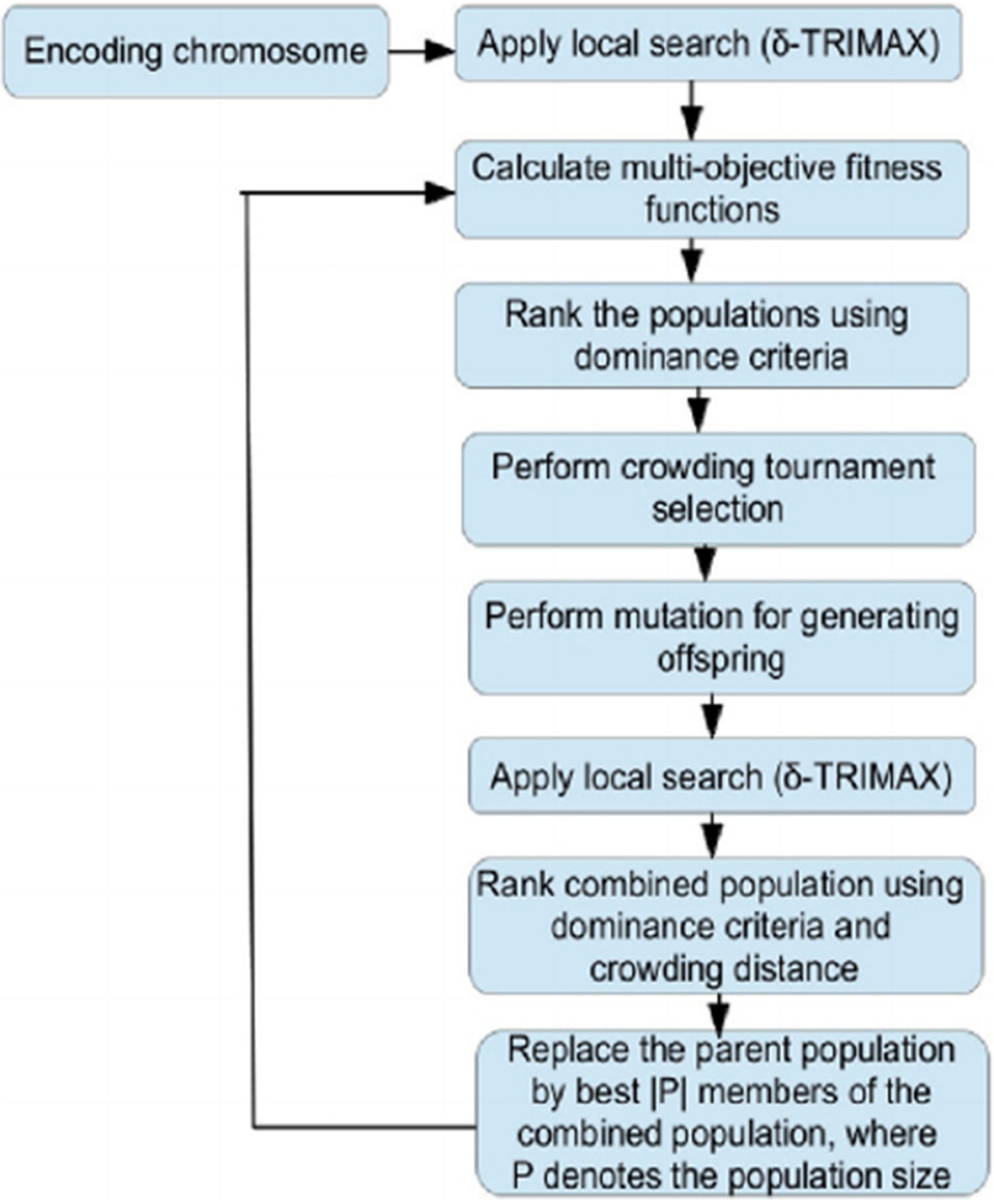
EMOA- *δ*-TRIMAX algorithm. Steps of the EMOA- *δ*-TRIMAX algorithm

The steps the of *δ*-TRIMAX algorithm [8, 9] have been described in the Additional file 1. Figure 2 shows the steps of our proposed EMOA- *δ*-TRIMAX algorithm.

#### Multi-objective optimization problem

The multi-objective optimization problem is equivalent to finding the vector $\bar {x}^{*} = [x_{1}^{*},x_{2}^{*},\ldots,x_{n}^{*}]^{T}$ of decision variables that satisfies a number of equality and inequality constraints by optimizing the vector function $\bar {f}(\bar {x}) = [f_{1}(\bar {x}),f_{2}(\bar {x}),\ldots,f_{r}(\bar {x})]^{T}$ subject to some constraints. Here the constraints correspond to the feasible region *F* that holds all the acceptable solutions; $\bar {x}^{*}$ stands for an optimal solution. For a minimization problem, Pareto optimality can be formally delineated as: A decision vector $\bar {x}^{*}$ is referred to as Pareto optimal if and only if there is no $\bar {x}$ such that ∀*i*∈{1,2,..,*r*}, $f_{i}(\bar {x}) \leq f_{i}(\bar {x}^{*})$ and ∃*i*∈{1,2,…,*r*}, $f_{i}(\bar {x}) \textless f_{i}(\bar {x}^{*})$. In words, $\bar {x}^{*}$ is called Pareto optimal if there exists no possible vector $\bar {x}$ that induces a diminution of some criterion without a contemporaneous increase of at least one other criterion [11, 14].

#### Genetic algorithm

A genetic algorithm is a search heuristic that imitates the process of Darwinian evolution [11, 14]. Here the population is generated randomly and consists of a set of chromosomes that encode the parameters of the search space. A fitness function corresponds to the objective function to be optimized and is used to estimate the goodness of each chromosome in the population. Genetic operators such as selection, crossover and mutation are used to evolve subsequent generations. If some particular criterion is met or the maximum generation limit is reached, then the algorithm finishes its execution.

#### Encoding chromosome

Each chromosome is represented by a binary string that has three parts. A chromosome encodes a possible tricluster. For a time series gene expression dataset having G number of genes, C number of samples and T number of time points, the first G bits correspond to genes, the next C bits represent the samples and the last T positions stand for the time points. Hence each string is represented by (G+C+T) bits, having a value either 1 or 0. A value 1 means the corresponding gene or sample or time point is a member of the tricluster. Suppose for a 3D gene expression dataset having 10 genes, 5 samples and 8 time points, a string {10010011100011101010101} represents that genes {*g*_1_,*g*_4_,*g*_7_,*g*_8_,*g*_9_}, samples {*s*_3_,*s*_4_,*s*_5_} and time points {*t*_2_,*t*_4_,*t*_6_,*t*_8_} are the members of the tricluster. The initial population consists of a set of randomly generated chromosomes. Retrieval of overlapping genes belonging to several triclusters are guaranteed by the step of chromosome encoding. As each bit of a chromosome in the population represents the presence or absence of genes, replicates and time points in one resultant tricluster, often we could find an overlap between the positions of any two chromosomes containing a value 1. Thus different chromosomes can encode overlapping triclusters. Some genes and/ or samples and/ or time points could be added to the initial population inspite of lying far away from the feature space. To remove such nodes from the population, *δ*-TRIMAX has been used as a local search heuristic.

#### Objective functions

After applying *δ*-TRIMAX as a local search heuristic, each string in the population represents one *δ*-tricluster having an MSR below a threshold *δ*. Now we compute values of the following three objective functions for each individual in the population. The first objective function is

(2) $$ f_{1} = \frac{MSR}{\delta},  $$

where MSR is the mean squared residue score of one tricluster. Hence, *f*_1_ needs to be minimized. The second objective function is

(3) $$ f_{2} = \frac{\left|I\right| \ast \left|J\right| \ast \left|K\right|}{\left|G\right| \ast \left|C\right| \ast \left|T\right|},  $$

where (|*I*|∗|*J*|∗|*K*|) is the volume of the tricluster and (|*G*|∗|*C*|∗|*T*|) is the volume of the dataset. Our goal is to maximize the value of *f*_2_. Finally the third objective function is

(4) $$ f_{3} = \left| 1-\frac{6\sum {d_{i}^{2}}}{n(n^{2}-1)} \right|,  $$

where *d*_*i*_ is the difference between the ranks of average expression values (sorted either in ascending or descending order) over a subset of samples at *i*th time point of each pair of genes in one tricluster and *n* is the number of time points in that tricluster. Here the goal is to maximize the non-parametric Spearman correlation coefficient (*f*_3_) [15] of the resultant triclusters.

#### Motivations of objective functions

As the aim of our proposed algorithm is to find triclusters having a lower MSR score and a higher volume, the first two objective functions (*f*_1_ and *f*_2_) ensure to accomplish those goals. Moreover the objective function *f*_3_ is used to maximize the correlation coefficients among genes belonging to the resultant triclusters. We have taken the absolute values of the correlation coefficients just considering the fact that coregulated genes can be both up- and down-regulated by the transcription factors across a subset of time points.

#### Genetic operators

Here, non-dominated sorting and crowding distance are used for fitness assignment and comparison [11]. A crossover is a generalization of several mutations performed at once, which we have not applied in this work [16]. Instead, we have used bit string mutations with a high mutation probability to generate offspring population from a parent population. In this case, the mutation occurs at random positions through bit flips. For instance, for a binary string {1011010010} we generate a random number ranges from 0 to 1 for each bit of the string. If this random number for a particular bit is less than or equal to the mutation probability, mutation occurs and the value 1 or 0 is changed to a value 0 or 1, respectively. The mutation probability remains same for each of the bits of chromosome. After applying the mutation operator on each individual of the population, some genes/samples/time points can be added to the population that are lying far away from the feature space. To cope with this problem we have applied *δ*-TRIMAX as a local search heuristic.

#### Elitism

We have included elitism to keep track of non-dominated Pareto optimal solutions after each generation [11]. Stopping criteria is measured by the convergence metric delineated in equation (8).

### Tricluster eigengene

We applied the singular value decomposition method (SVD) on the expression data of each resultant tricluster to detect its eigengene [17]. For instance, $X^{i}_{g \times (c \ast t)}$ stands for the expression matrix of *i*th tricluster, where g, c and t represent the number of genes, samples and time points of *i*th tricluster. Now we apply SVD on the data matrix (normalized to mean=0 and variance=1). Now, the SVD of *i*th tricluster can be represented as,

(5) $$ X^{i} = UDV^{T},  $$

where U and V are the orthogonal matrices. *U* ^*i*^ is a g ∗ (c ∗ t) matrix with orthonormal columns, *V* ^*i*^ is a (c ∗ t) × (c ∗ t) orthogonal matrix and *D* ^*i*^ is (c ∗ t) × (c ∗ t) diagonal matrix of singular values. Assuming that singular values in matrix *D* ^*i*^ are arranged in non-decreasing order, we can represent the eigengene of the *i*th tricluster by the first column of matrix *V* ^*i*^, i.e.

(6) $$ E^{i} ={V^{i}_{1}}.  $$

### KEGG pathway enrichment

To establish the biological significance of the genes belonging to each resultant tricluster for both datasets we have performed a KEGG pathway enrichment analysis using the GOStats package in R with a p-value cutoff (BH-corrected p-value) of 0.05 [18, 19].

### TFBS enrichment analysis

Genes that exhibit similar expression profiles are supposed to be regulated by the same mechanism. To analyze the potential co-regulation of co-expressed genes, we have done a transcription factor binding site (TFBS) enrichment analysis using the TRANSFAC library (version 2012.2) [20]. Here we used 52 million TFBS predictions that are conserved between human, mouse, dog and cow [21]. Out of these 52 million conserved TFBSs we have selected the highest-scoring 1 % for each TRANSFAC matrix to identify the most specific regulator (transcription factor) - target interactions. We have applied a hypergeometric test and Benjamini Yekutieli-FDR for p-value correction to find over-represented binding sites (p-value ≤ 0.05) in the upstream regions of genes belonging to each tricluster [22, 23].

### Datasets

#### Description of the artificial datasets

##### Artificial dataset 1 (AD1):

First, we have applied the proposed algorithm to an artificial dataset containing 1000 genes, 5 samples and 4 time points. We have then embedded 3 perfect shifting triclusters (standard deviation (*σ*)=0) of size 100 × 4 × 4, 80 × 4 × 4 and 60 × 4 × 4 into the dataset. In the next step, we have implanted 3 noisy triclusters with different levels of noise (*σ*=0.1,0.3,0.5,0.7,0.9) into the synthetic dataset.

##### Artificial dataset 2 (AD2):

Moreover, we have generated another artificial dataset which contains 200 genes, 10 replicates and 10 time points. Afterwards, we have implanted 3 perfect shifting triclusters (standard deviation (*σ*)=0) of size 50 × 3 × 3, 50 × 3 × 3 and 50 × 3 × 3 into the dataset. In the next step, we have added different levels of noise (*σ*=0.1,0.3,0.5,0.7,0.9) into the synthetic dataset.

##### Artificial dataset 3 (AD3):

To evaluate the performance of the proposed algorithm in case of the datasets containing different number of time points, we have generated three additional artificial datasets of size 200 (genes) × 10 (replicates) × 20 (time points), 200 (genes) × 10 (replicates) × 25 (time points) and 200 (genes) × 10 (replicates) × 30(time points) in which we have embedded 3 perfect shifting triclusters of size 30 × 3 × 8, 30 × 3 × 6 and 30 × 3 × 4.

##### Artificial dataset 4 (AD4):

In order to show the performance of the algorithm for the dataset containing missing values, we have randomly deleted the values of 0.5 *%*, 1 *%*, 1.5 *%* and 2 *%* of all elements of one artificial dataset of size 200 × 10 × 20 containing three triclusters of size 30 × 3 × 8, 30 × 3 × 6 and 30 × 3 × 4.

#### Description of real-life datasets

##### Dataset 1:

In this work, this previously published dataset has only been used for comparing the performance of the proposed algorithm with that of the other existing triclustering algorithms since one of the algorithms we wanted to compare our approach with, OPTricluster, can only be efficiently applied to a short time series gene expression dataset and thus, was not suitable to be used for dataset 2 (see below) [7]. Dataset 1 holds 54675 Affymetrix human genome U133 plus 2.0 probe ids, 3 samples and 4 time points (0, 3, 6 and 12 hours) (GSE11324) [24]. The goal of this experiment was to determine cis-regulatory sites in previously uncharted genome regions, responsible for conveying estrogen responses, and to identify the cooperating transcription factors that also contribute to estrogen signaling in MCF7 breast cancer cells.

##### Dataset 2:

This dataset contains 48803 Illumina HumanWG-6 v3.0 probe ids, 3 replicates and 12 time points (days 0, 3, 7, 10, 14, 20, 28, 35, 45, 60, 90 and 120) (GSE35671) [12]. All these replicates are independent of each other. The aim of this study was to provide insights into the molecular regulation of hiPSC differentiation to cardiomyocytes.

##### Dataset 3:

This experiment was carried out to study the dynamics of expression profiles of 54675 Affymetrix human genome U133 plus 2.0 probe ids in response to IFN-beta-1b treatment across four time points over 6 patients (GSE46280) [25].

## Results and discussion

### Results on an artificial dataset

To evaluate the performance of the proposed algorithm on the artificial datasets described above (2.6.1), we have used the affirmation score [8, 9] defined as

(7) $$ { \fontsize{7.7}{6}\begin{aligned} SM^{*}(T_{im}, T_{res}) = \sqrt{SM^{*}_{G}(T_{im}, T_{res}) \times SM^{*}_{C}(T_{im}, T_{res}) \times SM^{*}_{T}(T_{im}, T_{res})}, \end{aligned}}  $$

where, *T*_*im*_ is the set of implanted triclusters, *T*_*res*_ represents the set of triclusters extracted by any triclustering algorithm, $SM^{*}_{G}(T_{\textit {im}}, T_{\textit {res}})$ is the average gene affirmation score, $SM^{*}_{C}(T_{\textit {im}}, T_{\textit {res}})$ is the average sample affirmation score and $SM^{*}_{T}(T_{\textit {im}}, T_{\textit {res}})$ is the average time point affirmation score of *T*_*res*_ with respect to *T*_*im*_. The value of *S* *M* ^∗^(*T*_*im*_,*T*_*res*_) ranges from 0 to 1. If *T*_*res*_=*T*_*im*_, then the affirmation score is 1.

**Fig. 3:**
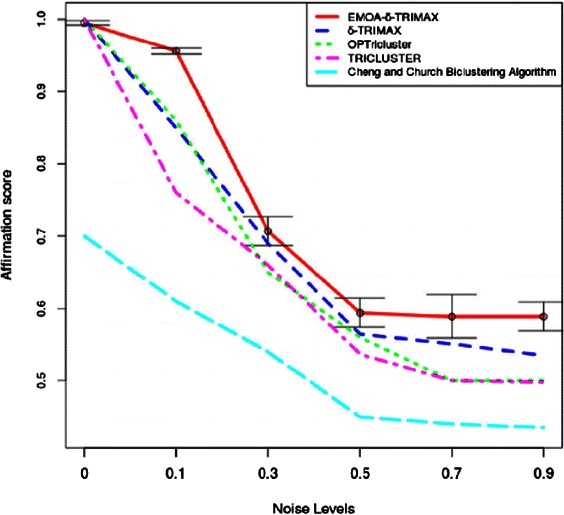
Comparison in terms of affirmation score. Comparison between EMOA- *δ*-TRIMAX, *δ*-TRIMAX, TRICLUSTER, OPTricluster and the biclustering algorithm proposed by Cheng and Church in terms of affirmation score for the artificial dataset 1

**Fig. 4:**
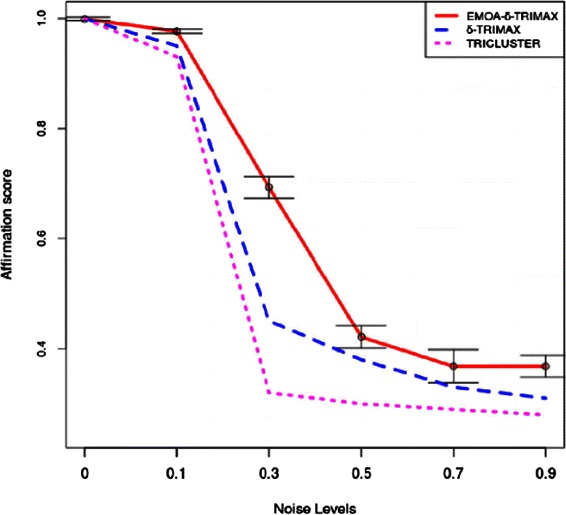
Comparison in terms of affirmation score. Comparison between EMOA- *δ*-TRIMAX, *δ*-TRIMAX and TRICLUSTER algorithm in terms of affirmation score for the artificial dataset 2

**Fig. 5:**
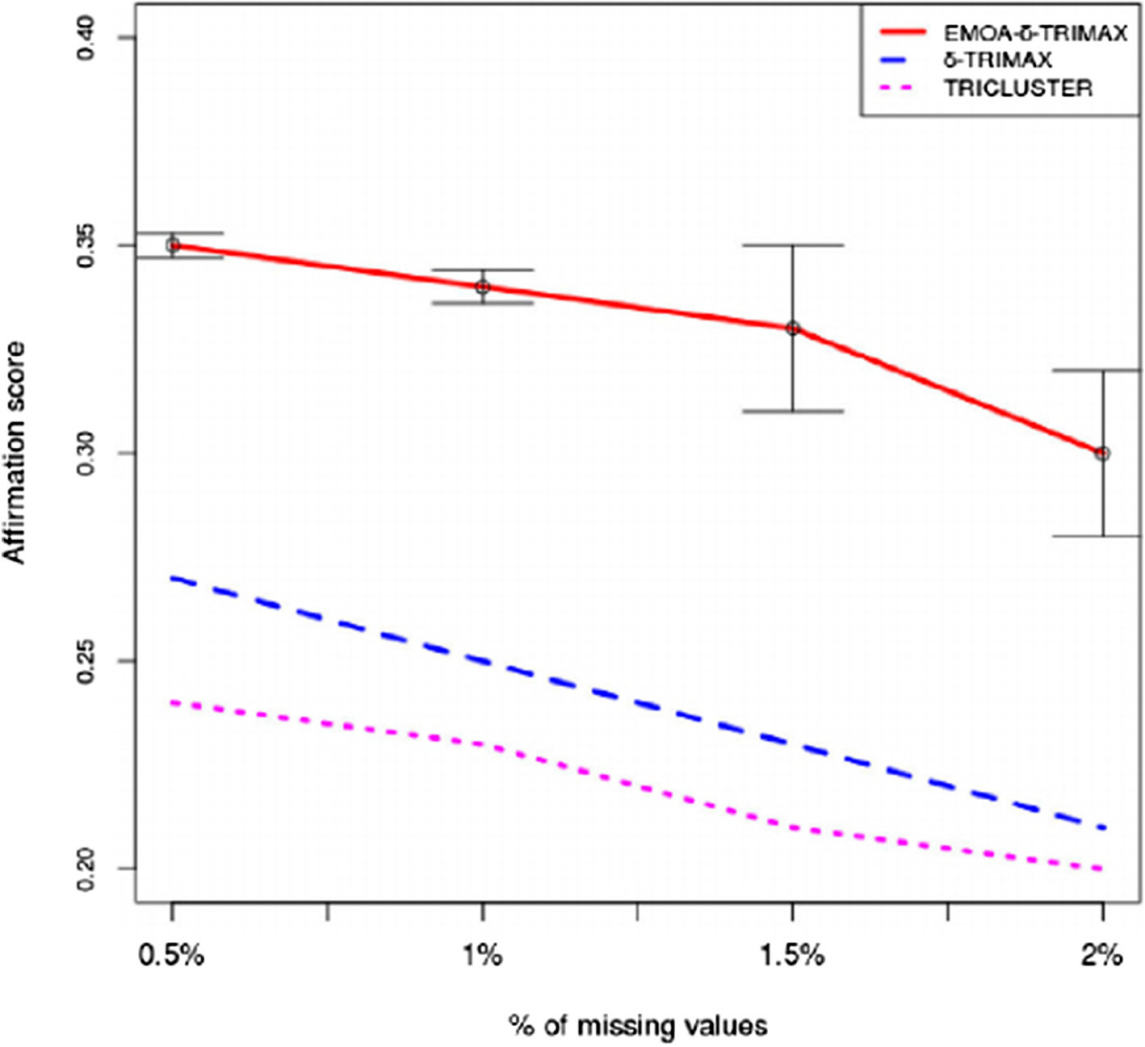
Comparison in terms of affirmation score. Comparison between EMOA- *δ*-TRIMAX, *δ*-TRIMAX and TRICLUSTER algorithm in terms of affirmation score for the artificial dataset 4

The affirmation score was also used to compare the performance of the proposed algorithm with that of the other triclustering algorithms and one biclustering algorithm [26]. Before applying the biclustering algorithm to the artificial dataset we have converted G × C × T dataset into a G × CT dataset. To compute the value of *δ*, we have first clustered the genes over all time points and then the time points over the subset of genes for each gene cluster in each sample plane using the k-means algorithm. Taking a randomly selected sample plane, we have computed the MSR score of the submatrix of each gene and time-point cluster and repeated this procedure 100 times. Then we have taken the lowest value as the value of *δ*. Although it is possible to minimize the MSR score without introducing the threshold parameter *δ*, minimizing MSR without using any threshold may either yield some small sized triclusters which may not provide any biologically meaningful information or produce large sized triclusters which may contain genes and/or samples and/or time points lying far apart from the feature space. Thus using a threshold parameter *δ* may balance the size and quality of the resultant triclusters. The value of *λ* has been experimentally set to maximize the speed of the proposed algorithm and minimize the risk falling into a local optimum. The values of *δ* and *λ* used to run the proposed algorithm and our previously proposed *δ*-TRIMAX algorithm on the artificial datasets are enlisted in Tables 1, 2, 3 and 4. We have tuned the input parameters of other triclustering algorithms rather than using the default parameter values in order to achieve better results on the artificial datasets. The OPTricluster algorithm, which was proposed to mine only short time series gene expression datasets and yields triclusters containing all the time points, has only been applied to the dataset AD1 as this dataset contains triclusters having the same number of time points as the entire dataset. For the rest of the artificial datasets used in this work, the time point affirmation scores will be deteriorated for OPTricluster algorithm which in turn affects the overall affirmation score. From Figs. 3 and 4 we can observe that the proposed algorithm outperforms the other algorithms for each of the artificial datasets in terms of affirmation score. Figure 5 shows that although the affirmation scores of the proposed algorithm become worse in case of the dataset containing missing data points, it still outperforms the other algorithms. Moreover, Table 5 indicates the fact that EMOA- *δ*-TRIMAX can effectively deal with the datasets having different number of time points.

**Table 1:** Values of input parameters of EMOA- *δ*-TRIMAX namely, *λ* and *δ* for different levels of noise in case of the artificial dataset 1 (AD1)

Noise levels (*σ*)	Values of *λ*	Values of *δ*
0	1.2	0.0002
0.1	1.2	0.025
0.3	1.2	0.115
0.5	1.2	0.26
0.7	1.2	0.49
0.9	1.2	0.85

**Table 2:** Values of input parameters of EMOA- *δ*-TRIMAX namely, *λ* and *δ* for different levels of noise in case of the artificial dataset 2 (AD2)

Noise Levels (*σ*)	Values of *λ*	Values of *δ*
0	1.2	0.00002
0.1	1.2	0.045
0.3	1.2	0.06
0.5	1.2	0.29
0.7	1.2	0.59
0.9	1.2	0.8

**Table 3:** Values of input parameters of EMOA- *δ*-TRIMAX namely, *λ* and *δ* for different levels of noise in case of the artificial dataset 3 (AD3_{a, b, c})

*λ* (AD3 _a)	*δ* (AD3 _a)	*λ* (AD3 _b)	*δ* (AD3 _b)	*λ* (AD3 _c)	*δ* (AD3 _c)
1.2	0.0002	1.2	0.02	1.2	0.02

**Table 4:** Values of input parameters of EMOA- *δ*-TRIMAX namely, *λ* and *δ* for different levels of noise in case of the artificial dataset 4 (AD4)

*%* of missing values	*λ*	*δ*
0.5	1.2	0.02
1	1.2	0.02
1.5	1.2	0.02
2	1.2	0.02

**Table 5:** Comparison between EMOA- *δ*-TRIMAX, *δ*-TRIMAX and TRICLUSTER algorithm in terms of affirmation score for the artificial dataset 3 (AD3 _a, AD3 _b, AD3 _c)

Dataset	EMOA- *δ*-TRIMAX	*δ*-TRIMAX	TRICLUSTER
AD3 _a	1	1	1
AD3 _b	1	1	1
AD3 _c	1	1	1

**Fig. 6:**
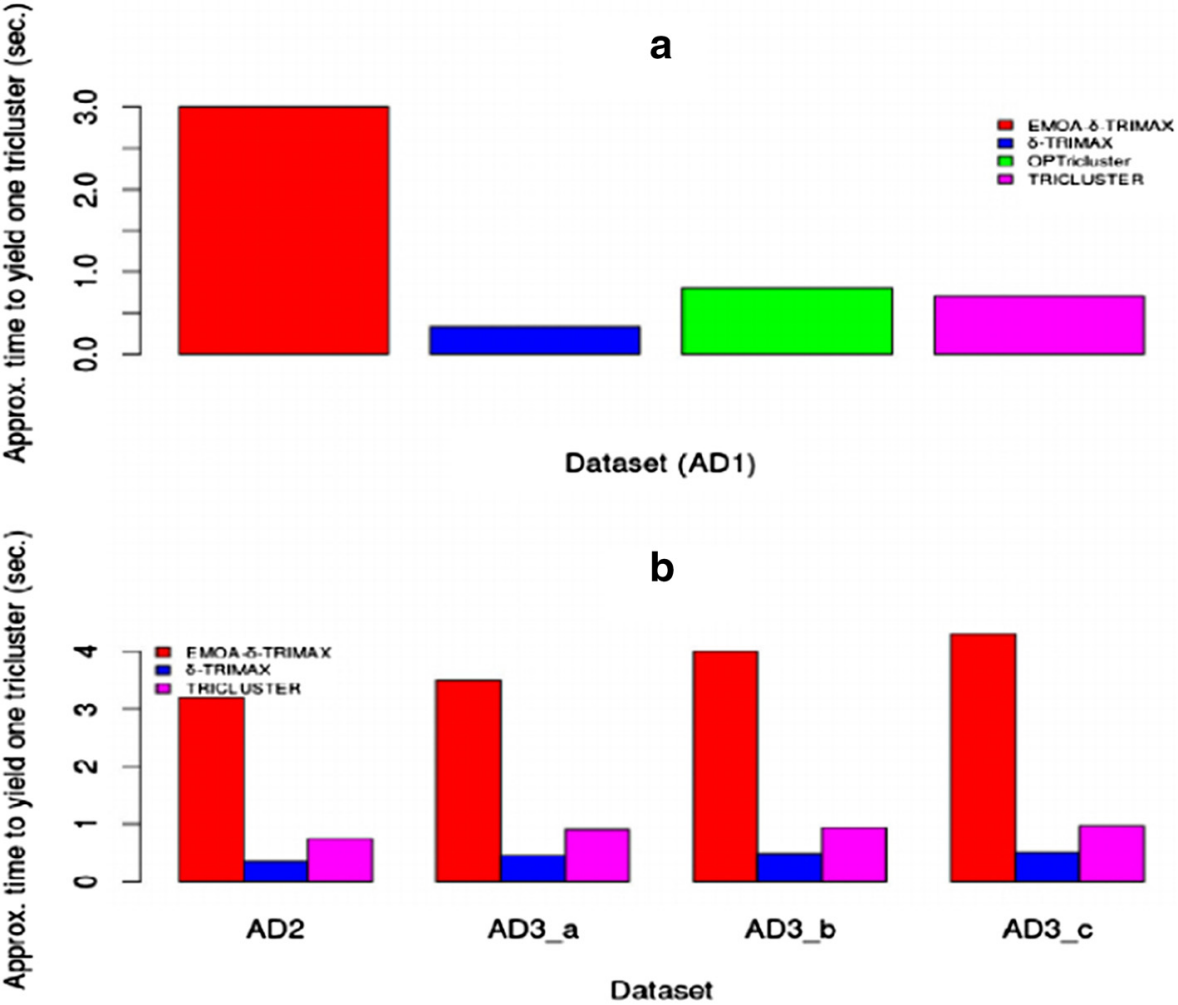
Comparison in terms of CPU time. Comparison between EMOA- *δ*-TRIMAX, *δ*-TRIMAX and TRICLUSTER algorithm in terms of CPU time for the artificial datasets 1 (**a**), 2 and 3 (**b**)

Moreover we compared the performance of the proposed algorithm with that of the existing ones in terms of CPU time. From Fig. 6, we can see that the proposed algorithm takes relatively more time to retrieve one tricluster as Non-dominated Sorting Genetic Algorithm-II (NSGA-II) has been used to optimize multiple objectives.

#### Robustness of the evolutionary algorithm

In order to show the robustness of the proposed algorithm, we have used the artificial datasets 1 and 2 with different levels of noise described above (2.6.1). For each of these datasets, we have run the proposed algorithm for 20 times and reported the standard deviations of the affirmation scores obtained after each run in Table 6 which establishes the robustness of the proposed algorithm as in case of each of the two datasets, the affirmation scores are very close to the mean.

**Table 6:** Standard deviations of the affirmation scores yielded by the EMOA- *δ*-TRIMAX algorithm for artificial dataset 1 (AD1) and 2 (AD2)

Noise levels (*σ*)	Standard deviation (AD1)	Standard deviation (AD2)
0	0.05	0.003
0.1	0.05	0.004
0.3	0.02	0.02
0.5	0.005	0.02
0.7	0.003	0.03
0.9	0.004	0.02

### Results on real-life datasets

As a data preprocessing step, we have used robust multi-array average (RMA) method to normalize the datasets. The values of the input parameters of EMOA- *δ*-TRIMAX are provided in Table 7. We have set the values of *λ* and *δ* of EMOA- *δ*-TRIMAX and our previously proposed *δ*-TRIMAX algorithms for each of the real-life datasets according to our criteria explained in section ‘Results on an artificial dataset’. As using default parameter values may often produce poor results, the input parameters of other algorithms were tuned in order to obtain better results on each of the real-life datasets. Table 8 shows the percentage of probe ids, replicates and time points that are covered by the triclusters obtained with the proposed algorithm.

**Table 7:** Values of input parameters of EMOA- *δ*-TRIMAX for each of the real-life datasets

Datasets	Dataset 1	Dataset 2	Dataset 3
*λ*	1.2	1.2	1.2
*δ*	0.012382	0.008	0.008754
Number of generations	100	100	100
Population Size	100	100	100
Mutation probability	0.9	0.9	0.9

**Table 8:** Percentage of probe ids, replicates and time points covered by the resultant triclusters for each of the real-life datasets

Datasets	Dataset 1	Dataset 2	Dataset 3
Coverage of probe ids	99.02 *%*	88.14 *%*	93 *%*
Coverage of replicates	100 *%*	100 *%*	100 *%*
Coverage of time points	100 *%*	100 *%*	100 *%*

### Convergence of solutions

**Fig. 7:**
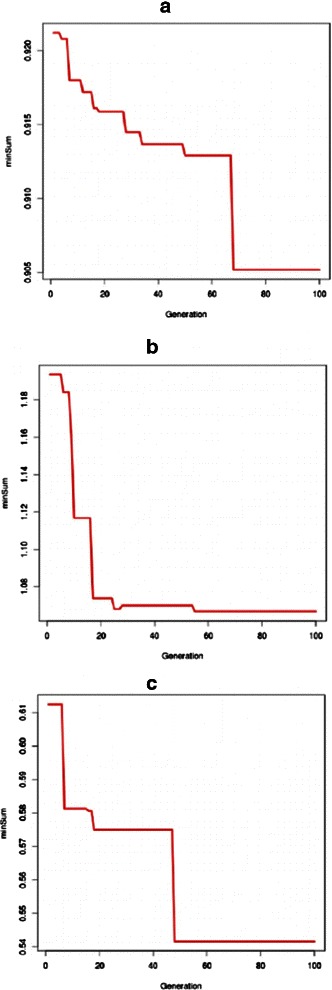
Convergence of solutions. Convergence of solutions towards the Pareto optimal front. *minSum* values are plotted for Dataset 1 (**a**), Dataset 2 (**b**) and Dataset 3 (**c**)

In order to show the convergence of solutions towards the Pareto optimal front around its center region, we have computed minSum values in each generation as follows

(8) $$ minSum(\Psi) = \min_{x \in \Psi} (f_{1}(x) + (1-f_{2}(x)) + (1-f_{3}(x))),  $$

where *Ψ* denotes the current population and *f*_1_, *f*_2_ and *f*_3_ correspond to the objective functions defined in section ‘Methods’. We have found that the solutions converge towards a Pareto optimal front in case of each of the real-life datasets (Fig. 7).

### Performance comparison

**Fig. 8:**
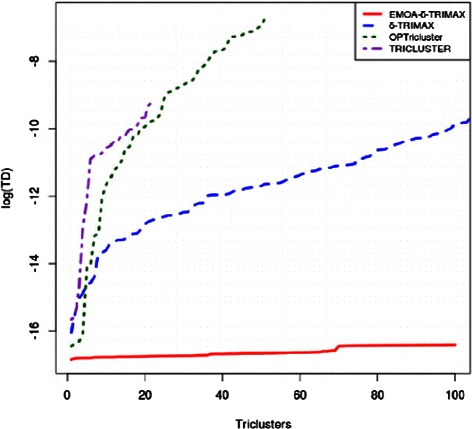
Comparison in terms of Tricluster Diffusion score for Dataset 1. Performance comparison between EMOA- *δ*-TRIMAX, *δ*-TRIMAX, TRICLUSTER and OPTricluster in terms of TD (in log scale) score for Dataset 1

**Fig. 9:**
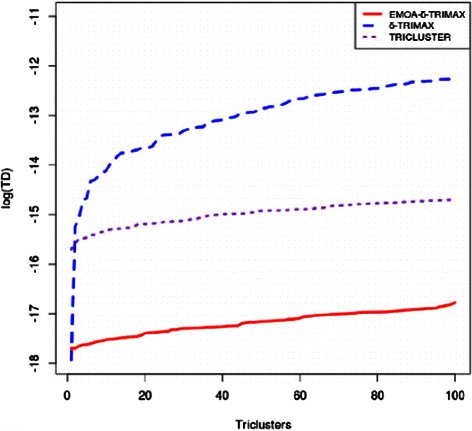
Comparison in terms of Tricluster Diffusion score for Dataset 2. Performance comparison between EMOA- *δ*-TRIMAX, *δ*-TRIMAX and TRICLUSTER in terms of TD (in log scale) score for Dataset 2

**Fig. 10:**
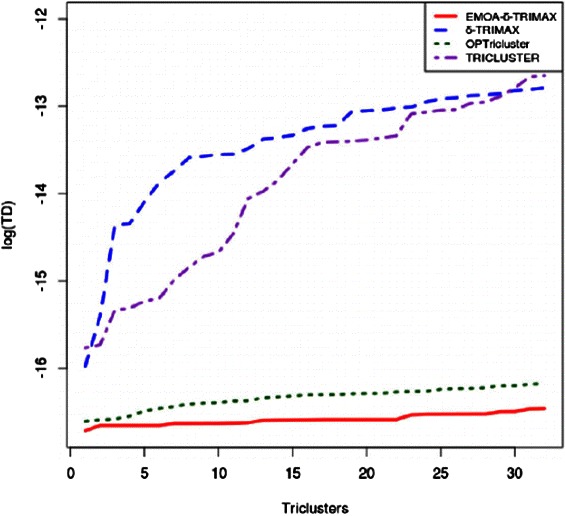
Comparison in terms of Tricluster Diffusion score for Dataset 3. Performance comparison between EMOA- *δ*-TRIMAX, *δ*-TRIMAX, TRICLUSTER and OPTricluster in terms of TD (in log scale) score for Dataset 3

We have applied our proposed algorithm on the three aforementioned real-life datasets and compared its performance with that of other triclustering algorithms. For this comparison, we have computed a Tricluster Diffusion (TD) score and a Statistical Difference from Background (SDB) score [27]. The TD score has been defined by equation 9.

(9) $$ TD_{i} = \frac{MSR_{i}}{Volume_{i}},  $$

where *M* *S* *R*_*i*_ and *V* *o* *l* *u* *m* *e*_*i*_ stand for the mean squared residue score (see eq. (1)) and for the volume of each resultant tricluster *i*. The volume of the *i*th tricluster can be defined as (|*I*_*i*_|∗|*J*_*i*_|∗|*K*_*i*_|), where |*I*_*i*_|, |*J*_*i*_| and |*K*_*i*_|, represent the number of genes, samples and time points of the *i*th tricluster, respectively. A lower TD score represents better quality of triclusters. Figures 8, 9 and 10 plot the TD scores (in log scale) of the resultant triclusters produced by all algorithms, showing that EMOA- *δ*-TRIMAX yields triclusters having lower TD scores than those produced by other algorithms for each of the three datasets.

The statistical difference from background (SDB) score signifies whether a set of n triclusters is statistically different from the background data matrix. The SDB score is defined by equation 10. A higher SDB score signifies better performance of the algorithm.

(10) $$ SDB = \frac{1}{n}\sum_{i=1}^{n}\frac{\frac{1}{r}\sum_{j=1}^{r}RMSR_{j}-MSR_{i}}{MSR_{i}},  $$

where *n* is the total number of triclusters extracted by the algorithm. *M* *S* *R*_*i*_ represents the mean squared residue of the *i*th tricluster retrieved by the algorithm and *R* *M* *S* *R*_*j*_ stands for the mean squared residue of the *j*th random tricluster having the same number of genes, experimental samples and time points as the *i*th resultant tricluster. Here a higher value of the numerator indicates a better quality of the resultant tricluster. In our study we have set *r* to 100. OPTricluster can not be applied to Dataset 2 as it effectively mines only short time series gene expression data having approximately 3-8 time points. From Tables 9, 10 and 11 we can observe the highest SDB scores for EMOA- *δ*-TRIMAX algorithm in case of the dataset 1, dataset 2 and dataset 3.

**Table 9:** Performance comparison between EMOA- *δ*-TRIMAX, *δ*-TRIMAX, TRICLUSTER and OPTricluster in terms of SDB score for Dataset 1

Algorithms	SDB score
EMOA- *δ*-TRIMAX	2.49851
*δ*-TRIMAX	2.140935
TRICLUSTER	2.094091
OPTricluster	0.4956035

**Table 10:** Performance comparison between EMOA- *δ*-TRIMAX, *δ*-TRIMAX and TRICLUSTER in terms of SDB score for Dataset 2

Algorithms	SDB score
EMOA- *δ*-TRIMAX	13.88559
*δ*-TRIMAX	12.10529
TRICLUSTER	7.520363

**Table 11:** Performance comparison between EMOA- *δ*-TRIMAX, *δ*-TRIMAX, TRICLUSTER and OPTricluster in terms of SDB score for Dataset 3

Algorithms	SDB score
EMOA- *δ*-TRIMAX	9.454915
*δ*-TRIMAX	8.945816
TRICLUSTER	7.076184
OPTricluster	0.4383489

### Biological significance

KEGG pathway enrichments have been found for each resultant tricluster for datasets 1, 2 and 3. To compare the performance of our proposed algorithm with that of the other algorithms using KEGG pathway enrichment we used a hit score [28]. The hit score for KEGG pathway enrichment can be delineated by equation 11.

(11) $$ Hit(K) = \frac{\max \{ \left|{N^{1}_{T}}\right|,\left|{N^{2}_{T}}\right|,\ldots,\left|{N^{n}_{T}}\right| \}}{\left|T\right|},  $$

where ${N^{i}_{T}}$ is the intersection gene set of tricluster *T* and its enriched KEGG pathway term *i*; |*T*| is the total number of genes in tricluster *T*. A higher hit score signifies that more genes in *T* participate in a canonical pathway.

We have observed TFBS enrichment for 98 *%*, 96 *%* and 94 *%* of all resultant triclusters for datasets 1, 2 and 3, respectively. We used a Hit score (equation (12)) to compare the performance of EMOA- *δ*-TRIMAX with that of other triclustering algorithms using the results of TFBS enrichment.

(12) $$ Hit(TF) = \frac{\max \{\left|{P^{1}_{T}}\right|,\left|{P^{2}_{T}}\right|,\ldots,\left|{P^{n}_{T}}\right| \} }{\left|T\right|},  $$

where ${P^{i}_{T}}$ is the intersection gene set of tricluster *T* and its enriched TRANSFAC matrix *i*; |*T*| is the total number of genes in tricluster *T*. A higher hit score signifies that more genes in *T* are regulated by a common transcription factor.

**Fig. 11:**
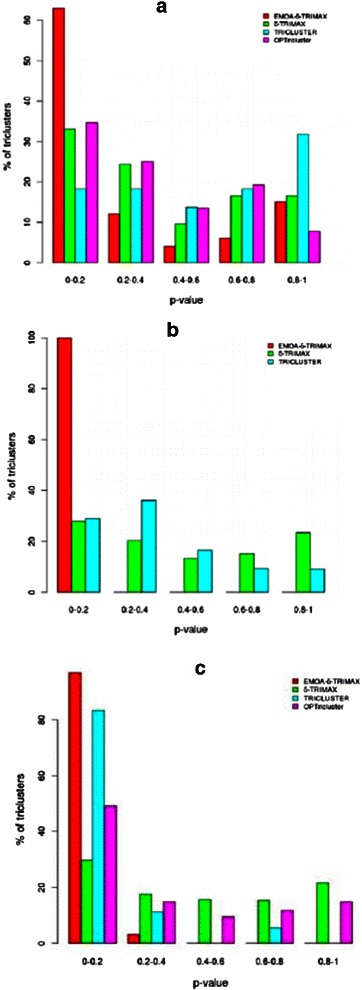
Comparison in terms of Hit score using KEGG pathway enrichment. Performance comparison between EMOA- *δ*-TRIMAX, *δ*-TRIMAX, TRICLUSTER and OPTricluster in terms of Hit scores for Datasets 1 (**a**), 2 (**b**) and 3 (**c**)

**Fig. 12:**
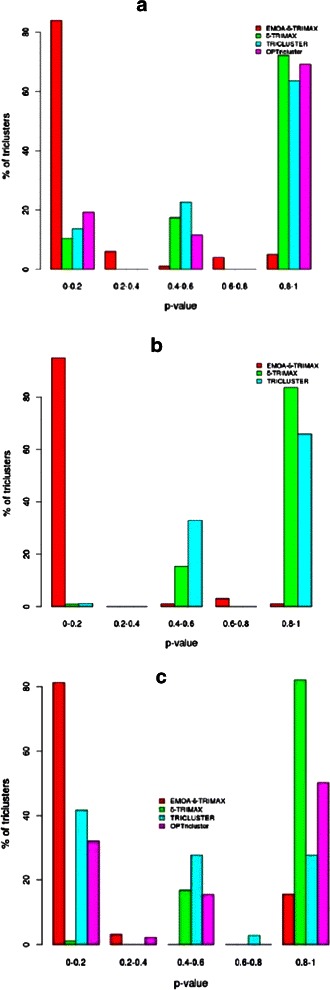
Comparison in terms of Hit score using TFBS enrichment. Performance comparison between EMOA- *δ*-TRIMAX, *δ*-TRIMAX, TRICLUSTER and OPTricluster in terms of Hit scores for Datasets 1 (**a**), 2 (**b**) and 3 (**c**)

At first we have calculated the hit scores Hit(K) and Hit(TF) for each resultant tricluster using KEGG pathway and TFBS enrichment results, respectively. For each tricluster (T) we generated 100 random gene lists having the same size as the tricluster (T). The Hit scores for each randomly generated gene list were computed using KEGG pathway and TFBS enrichment results. As final step we have applied the non-parametric Mann-Whitney-Wilcoxon test to compute the significance between these two sets of hit scores in terms of p-values [29]. From Figs. 11 and 12 it can be seen that EMOA- *δ*-TRIMAX yields more significant triclusters than the other algorithms in terms of hit scores computed from the enriched KEGG pathways and TRANSFAC matrices for each of the real-life datasets because a higher percentage of triclusters obtained by the proposed algorithm have a smaller p-value than those produced by the other algorithms for each of the real-life datasets. Particularly striking is the inverse trend of Hit Scores in the TFBS enrichment observed with EMOA- *δ*-TRIMAX, which has by far the largest population at the lowest p-values, and the other algorithms, where an increasing number of clusters is found with increasing p-values (Fig. 12 a–c).

### Importance of clustering biological replicates in 3D gene expression datasets

**Fig. 13:**
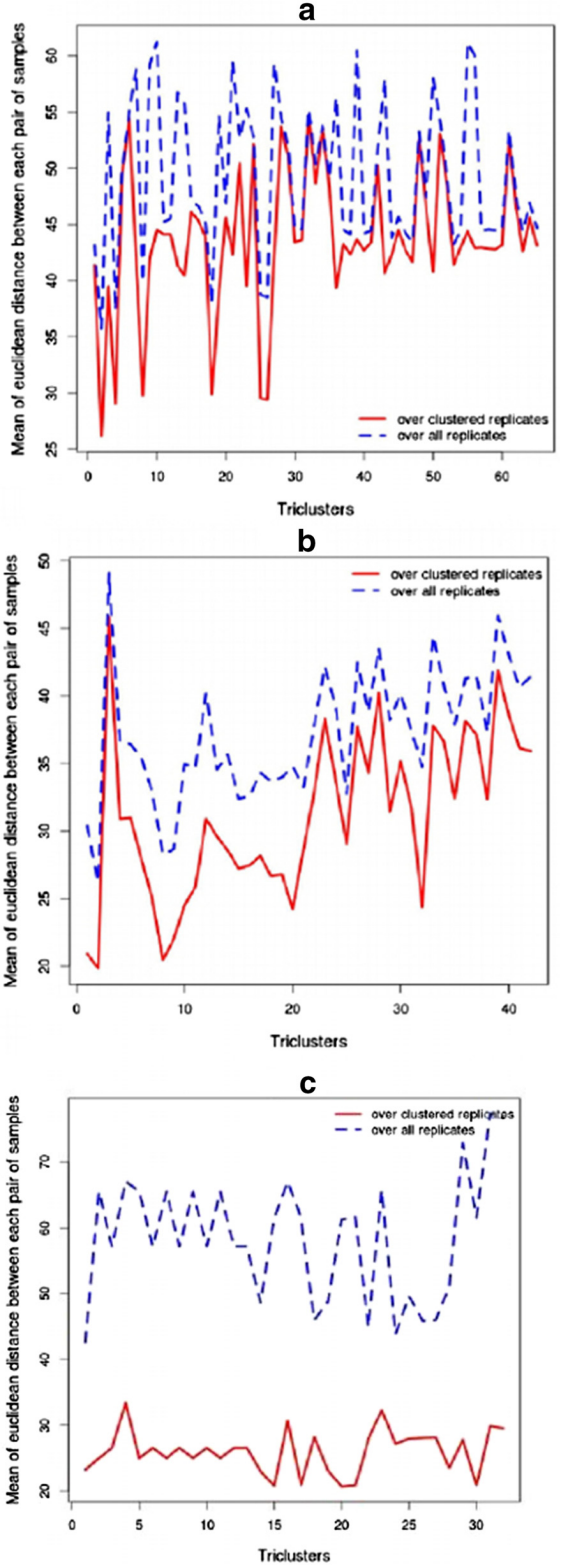
Importance of clustering replicates. Average Euclidean distances between the expression profiles of each pair of clustered (red) and all (blue, dashed line) replicates over the clustered genes and time points for Dataset 1 (**a**), Dataset 2 (**b**) and Dataset 3 (**c**)

Time series microarray experiments are performed to measure the expression profiles of genes at a set of time points. At each time point, the experiments are often repeated for a certain number of times, which in turn yield the expression profiles of the genes over a set of biological replicates at each time point. Though the expression profiles of these biological replicates are measured at the same time point keeping the experimental setup unchanged, peculiarities in experimental protocol or physiological variation of the population may cause disparity in the expression profiles of technical or biological replicates, respectively. Thus grouping those replicates which exhibit similar expression profiles might play an important role to identify those replicates that behave similarly. This enables us to retrieve biologically meaningful information from these samples rather than leveling effects by forcing together samples exhibiting dissimilar expression profiles. Here, we have tried to unravel the reason of not always getting all the replicates as the members of each resultant tricluster. In Fig. 13, we have plotted the mean of the Euclidean distances between the expression profiles of each pair of clustered samples over the clustered genes and time points along with that of each pair of all replicates. From this figure, we can notice the enhancement of the average intra-cluster distances between replicates while incorporating the missing replicates into our resultant triclusters for each of the real-life datasets. Thus, grouping the closest biological replicates improves the quality of the resultant triclusters and thus may play instrumental roles in extracting more biologically meaningful information. From Fig. 13 we can see that Dataset 3 has the most divergent replicates. It is not astonishing to us as in Dataset 1, the expression profiles were measured during the response of a well-controlled cell culture to estrogen; in Dataset 2, the expression profiles were monitored during the development differentiation of a cell culture over a long time period, with many chance to diverge whereas, in case of Dataset 3, replicates correspond to six human individuals where the applied interferon-beta elicited highly divergent responses.

### Identifying key genes of triclusters and analyzing their roles during hiPSC differentiation into cardiomyocytes

**Fig. 14:**
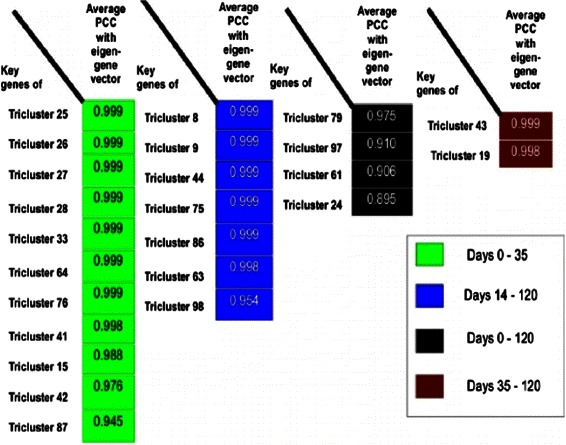
Average Pearson correlation coefficient (PCC) between key genes. Average Pearson correlation coefficient (PCC) between 10 top-most probe ids of triclusters and the corresponding eigen-gene vectors during different phases of cardiomyocyte differentiation

**Fig. 15:**
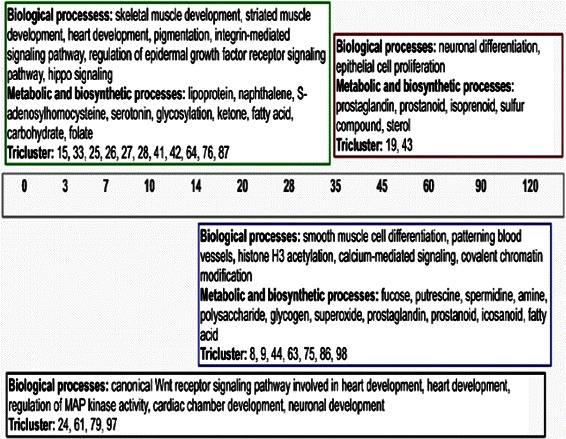
Summarizations of GOBPs and metabolic pathways of the key genes of resultant triclusters. Summarization of enriched GOBPs and metabolic pathways of the key genes of the mentioned triclusters during hiPSC differentiation to cardiomyocytes. Green, red, blue and black colored boxes represent the time points Days 0 to 35, Days 35 to 120, Days 14 to 120 and Days 0 to 120, respectively

To detect key genes, we have first represented each tricluster by its eigen-gene and then computed the Pearson correlation coefficient between each gene of the tricluster and its eigen-gene. We then ranked the probe-ids in descending order of Pearson correlation coefficient. We consistently observed that the genes corresponding to, for instance the 10 top-most probe-ids exhibited clear functional characteristics with relevance for cardiac development (or concomitant processes, see below) when being mapped to GOBPs (Gene Ontology Biological Processes) or metabolic pathways (from KEGG). Therefore, we considered them as “key genes” of that tricluster. Usually, no similarly clear categorizations were found for all the genes of one tricluster. For instance, if we perform biological process enrichment test using all genes of tricluster 64, we would not find the biological processes like S-adenosylhomocysteine, lipoprotein metabolic processes as the enriched ones. From Fig. 14 we can see that the identified key genes of the triclusters are highly correlated with the corresponding eigen-gene vectors. It has been stated in the original work that cardiomyocyte differentiation (dataset 2) occurs during days 0, 3, 7, 10, 14, 20, 28, 35 whereas days 35, 45, 60, 90, 120 are the post-differentiation time points [12]. On day 14, the heart beating was first observed. Figure 15 summarizes the corresponding GOBPs (Gene Ontology Biological Process) and metabolic pathways of the corresponding tricluster key genes during different stages of cardiomyocytes differentiation. It is of interest to observe enrichment of several biosynthetic and metabolic processes such as lipoprotein, naphthalene, S-adenosylhomocysteine, serotonin, fucose, putrescine, ketone, prostanoid, fatty acid, carbohydrate, spermidine etc. and amine, putrescine, folate biosynthetic processes during stem cell differentiation into cardiomyocyte. Each of the aforementioned metabolic and biosynthetic processes is known to play an instrumental role in either heart development or in preventing cardiovascular diseases [30–41]. Moreover, the enriched biological processes show the parallel occurrence of neural and cardiac development [42]. This is not too surprising since a previous study reported that the crosstalk between the neuronal and the cardiovascular system may play a pivotal role in the development of both systems [43]. The lists of enriched processes also reveal the occurrences of smooth, cardiac and skeletal muscle cell differentiations during cardiomyocyte development; this finding is also supported by previous reports [44]. Moreover, the instrumental role of the canonical Wnt receptor signaling pathway involved in heart development can also be deduced from the list of enriched biological processes involved in all stages of differentiation. A previous study inferred Wnt signaling pathway as an important regulator during cardiomyocyte differentiation [45]. Furthermore, through our analysis we have identified the enrichment of biological process such as histone H3 acetylation or the hippo signaling which are also inferred to be functionally associated with the characteristics of hiPSC-derived cardiomyocytes [46, 47]. The Additional file 1: Tables S1-S5 contain the lists of enriched GOBPs/ KEGG pathways of the triclusters shown in Fig. 15. Additionally, in Additional file 1: Tables S6-S8, we have enlisted genes that are already known to play important roles in cardiovascular diseases and development, in addition to genes that are hypothesized to be functional in this context by interpreting and associating their general biological functions.

## Conclusion

In this work, we have shown that the improved version of our previously proposed triclustering algorithm EMOA- *δ*-TRIMAX outperforms the other algorithms when applied to four synthetic datasets as well as on three real-life datasets used in this work. Moreover, after retrieving groups of co-expressed and co-regulated genes over a subset of samples and across a subset of time points from a microarray gene expression dataset of hiPSC-derived cardiomyocyte differentiation, using the singular value decomposition method we have detected tricluster key genes most of which have already been shown or inferred to play instrumental roles in cardiac development. Thus, the other identified key genes can be hypothesized to be meaningful in this context as well, which needs to be experimentally validated. Furthermore, the enriched biological processes for the identified key genes of each tricluster not only resulted in a set of biological processes, associated with stem cell differentiation into cardiomyocytes but also a set of metabolic processes, the majority of which are known to play crucial roles in preventing cardiac diseases. Thus, the identified metabolic processes can be used to provide insights into potential therapeutic strategies to the treatment of cardiovascular diseases. Moreover, the triclusters for which the identified key genes are found to be involved in heart development might be facilitative to unravel regulatory mechanisms during different stages of cardiomyocyte development.

## Additional file

Additional file 1
**Algorithm I (** ***δ*** **-TRIMAX).**
