# Supplementary material for: Multiobjective triclustering of time-series transcriptome data reveals key genes of biological processes
Source: BMC Bioinformatics. 2015 Jun 26;16:200. doi: 10.1186/s12859-015-0635-8 (PMC4480927; doi:10.1186/s12859-015-0635-8)
Supplement: Additional file 1 — Algorithm I ( δ -TRIMAX). [file 12859_2015_635_MOESM1_ESM.pdf]

Additional file 1

Algorithm I (δ-TRIMAX):

Input. D, a matrix that represents 3D microarray gene expression dataset, $\lambda > 1$, an input parameter for multiple node deletion algorithm, $\delta \geq 0$, maximum allowable MSR score.
Output. All possible δ-triclusters.
Initialization. D' $\leftarrow$ D
Repeat
a. $D'_1 \leftarrow$ Results of Algorithm II on D' using δ and λ. If the no. of genes (conditions/samples and/or no. of time points) is 50 (This value can be chosen experimentally. Large value increases the execution time of the algorithm as it then executes more number of iterations.), then do not apply Algorithm II on genes (conditions/samples and/or time points).
b. $D'_2 \leftarrow$ Results of Algorithm III on D'_1 using δ.
c. $D'_3 \leftarrow$ Results of Algorithm IV on D'_2.
Until(No gene is found for δ-tricluster)

Algorithm II (Multiple node deletion):
This step uses a greedy search heuristic to delete nodes i.e genes/ samples/ time points from the 3D dataset to accomplish larger diminish of MSR score.
Input. D, a matrix of real numbers that represents 3D microarray gene expression dataset; $\delta \geq 0$, maximum allowable MSR threshold, $\lambda > 1$, threshold for multiple node deletion. The value of λ has been set experimentally to optimize the speed and performance (to avoid falling into local optimum) of the algorithm.
Output. M_{IJK}, a δ-tricluster, consisting of a subset(I) of genes, a subset(J) of samples/ experimental conditions and a subset of time points, having MSR score less than or equal to δ.
Initialization. I $\leftarrow$ {set of all genes}, J $\leftarrow$ {set of all experimental conditions/ samples} and K $\leftarrow$ {set of all time points} and to M(I,J,K) $\leftarrow$ D
Repeat
Calculate m_{iJK}, $\forall$ i $\in$ I; m_{IjK}, $\forall$ j $\in$ J; m_{IJk}, $\forall$ k $\in$ K; m_{IJK} and MSR.
If MSR $\leq \delta$ return M(I,J,K)
Else
Delete genes i $\in$ I that satisfy the following inequality

$$\frac{1}{|J||K|}\Sigma_{j\in J,k\in K}(m_{ijk} - m_{iJK} - m_{IjK} - m_{IJk} + 2m_{IJK})^2 > \lambda MSR$$

Recalculate m_{iJK}, $\forall$ i $\in$ I; m_{IjK}, $\forall$ j $\in$ J; m_{IJk}, $\forall$ k $\in$ K; m_{IJK} and MSR Delete samples/experimental conditions j $\in$ J that satisfy the following inequality

$$\frac{1}{|I||K|}\Sigma_{i\in I,k\in K}(m_{ijk} - m_{iJK} - m_{IjK} - m_{IJk} + 2m_{IJK})^2 > \lambda MSR$$

Recalculate m_{iJK}, ∀ i ∈ I; m_{IjK}, ∀ j ∈ J; m_{IJk}, ∀ k ∈ K; m_{IJK} and MSR Delete time points k ∈ K that satisfy the following inequality

$$\frac{1}{|I||J|}\Sigma_{i\in I,j\in J}(m_{ijk}-m_{iJK}-m_{IjK}-m_{IJk}+2m_{IJK})^2>\lambda MSR$$

End If
Until(There is no change in I, J and/or K)

Algorithm III (Single node deletion):
This step is optional. If the previous step yields triclusters that have MSR sore below a threshold δ, then there is no need to execute single node deletion algorithm.
Input. D, a matrix of real numbers that represents 3D microarray gene expression dataset; $\delta \geq 0$, maximum allowable MSR threshold.
Output. M_{IJK}, a δ-tricluster, consisting of a subset(I) of genes, a subset(J) of samples/experimental conditions and a subset of time points, having MSR score less than or equal to δ.
Initialization. I ← {set of all genes in D}, J ← {set of experimental conditions/samples in D} and K ← {set of time points in D} and to M(I,J,K) ← D

Calculate m_{iJK}, ∀ i ∈ I; m_{IjK}, ∀ j ∈ J; m_{IJk}, ∀ k ∈ K; m_{IJK} and MSR.
While MSR > δ Detect gene i ∈ I that has the highest score

$$\mu(i)=\frac{1}{|J||K|}\Sigma_{j\in J,k\in K}(m_{ijk}-m_{iJK}-m_{IjK}-m_{IJk}+2m_{IJK})^2$$

Detect sample/experimental condition j ∈ J that has the highest score

$$\mu(j)=\frac{1}{|I||K|}\Sigma_{i\in I,k\in K}(m_{ijk}-m_{iJK}-m_{IjK}-m_{IJk}+2m_{IJK})^2$$

Detect time point k ∈ K that has the highest score

$$\mu(k)=\frac{1}{|I||J|}\Sigma_{i\in I,j\in J}(m_{ijk}-m_{iJK}-m_{IjK}-m_{IJk}+2m_{IJK})^2$$

Delete gene or sample/experimental condition or time point that has highest μ score and modify I or J or K. Recalculate m_{iJK}, ∀ i ∈ I; m_{IjK}, ∀ j ∈ J; m_{IJk}, ∀ k ∈ K; m_{IJK} and MSR.
End while
Return M(I,J,K)

Algorithm IV (Node addition):
As multiple node deletion step uses greedy search approach to produce δ-triclusters, there may exist some nodes that can still be added without increasing the MSR score. So this step aims to maximize the volume of the resultant triclusters without increasing the MSR score.
Input. D, a matrix of real numbers that represents δ-tricluster, having a subset

of genes (I), a subset of experimental conditions/samples (J) and a subset of time points (K).
Output. $M_{I'J'K'}$, a δ-tricluster, consisting of a subset of genes (I') , a subset of samples/experimental conditions (J') and a subset of time points (K'), such that I $\subset$ I', J $\subset$ J', K $\subset$ K' and MSR(I',J',K') $\leq$ MSR of D.
Initialization. M(I,J,K) $\leftarrow$ D
Repeat
Calculate m_{iJK}, $\forall$ i; m_{IjK}, $\forall$ j; m_{IJk}, $\forall$ k; m_{IJK} and MSR.
Add samples/experimental conditions j $\notin$ J that satisfy the following inequality

$$\frac{1}{|I||K|}\Sigma_{i\in I,k\in K}(m_{ijk} - m_{iJK} - m_{IjK} - m_{IJk} + 2m_{IJK})^2 \leq MSR$$

Recalculate m_{iJK}, $\forall$ i; m_{IJk}, $\forall$ k; m_{IJK} and MSR
Add time points k $\notin$ K that satisfy the following inequality

$$\frac{1}{|I||J|}\Sigma_{i\in I,j\in J}(m_{ijk} - m_{iJK} - m_{IjK} - m_{IJk} + 2m_{IJK})^2 \leq MSR$$

Recalculate m_{IjK}, $\forall$ j; m_{IJk}, $\forall$; m_{IJK} and MSR
Add genes i $\notin$ I that satisfy the following inequality

$$\frac{1}{|J||K|}\Sigma_{j\in J,k\in K}(m_{ijk} - m_{iJK} - m_{IjK} - m_{IJk} + 2m_{IJK})^2 \leq MSR$$

Add genes i $\notin$ I that satisfy the following inequality

$$\frac{1}{|J||K|}\Sigma_{j\in J,k\in K}(-m_{ijk} + m_{iJK} - m_{IjK} - m_{IJk} + 2m_{IJK})^2 \leq MSR$$

Until(There is no change in I, J and/or K)
I' $\leftarrow$ I, J'$\leftarrow$ J, K' $\leftarrow$ K
Return I', J', K'

Table S1: Time points, samples, key genes of triclusters and the enriched gene ontology biological processes and/ or KEGG pathways during cardiomyocytes differentiation. Functions of bold gene symbols have been described in Tables S6-S8.

Tricluster	GOBP/ KEGG Pathway	Time Points	Samples	Key Genes
15	BP: GO:0045843: negative regulation of striated muscle tissue development (LUC7L), GO:0048635: negative regulation of muscle organ development (LUC7L), GO:0048634: regulation of muscle organ development (LUC7L), GO:0014706: striated muscle tissue development (LUC7L) ; KP: KEGG:00790: folate biosynthesis (DHFR), KEGG: 00670: one carbon pool by folate (DHFR)	3day-7day-10day-28day-35day	S1-S2-S3	**DHFR/ P1**, **GREB1**, ACTR3BP3, **ZNF365**, **SYN1**, PRM2, **LUC7L**
25	BP: GO:0035329: hippo signaling (TEAD1), GO:0070085: glycosylation (ABO), GO:0043413: macromolecule glycosylation (ABO); KP: KEGG:00601: Glycosphingolipid biosynthesis - lacto and neolacto series (ABO), KEGG:04270: Vascular smooth muscle contraction (GNA12)	0day-20day-35day	S1-S2	SNHG14, FABP5P11, **TEAD1**, EEF1A1P15, **PROKR2**, ABO, EVI2A, **BEST3**, GNA12
26	BP: GO:0042427: serotonin biosynthetic process (TPH2), GO:0042428: serotonin metabolic process (TPH2), GO:0007623: circadian rhythm (TPH2), GO:0043627: response to estrogen stimulus (TPH2)	3day-10day-14day	S2-S3	**TCTEX1D4**, ZG-PAT, OR9A4, **TPH2**, ENTPD5, MPP7, TMEM245, HTR5A
27	BP: GO:0045742: positive regulation of epidermal growth factor receptor signaling pathway (GPER), GO:0019722: calcium-mediated signaling (RGN), GO:0070374: positive regulation of ERK1 and ERK2 cascade (GPER), GO:0070371: ERK1 and ERK2 cascade (GPER), GO:0007173: epidermal growth factor receptor signaling pathway (GPER)	3day-14day-20day	S2-S3	ZRANB3, **NDUFAF6**, **RGN**, FAM163B, C18orf62, **GPER**, **FAIM2**, LETM2, NUB1, **EMR1**
28	BP: GO:0072366: regulation of cellular ketone metabolic process by positive regulation of transcription from RNA polymerase II promoter (PPARA), GO:0045923: positive regulation of fatty acid metabolic process (PPARA), GO:0006109: regulation of carbohydrate metabolic process (PPARA), GO:0042157: lipoprotein metabolic process (PPARA); KP: KEGG:04340: Hedgehog signaling pathway (WNT8A)	0day-3day-20day	S1-S2	MFSD6L, TTTY16, **WNT8A**, PAK7, **PPARA**, **EPHB2**, MRPL49, DCHS2, **CALML4**, ASB4
33	BP: GO:0060537: muscle tissue development (COL19A1), GO:0060538: skeletal muscle organ development (COL19A1), GO:0014706: striated muscle tissue development (COL19A1)	0day-10day-20day	S1-S2	ITGB5-AS1, SLC25A51P1, CLDN18, FAM90A6P, FAM90A7P, TTTY11, MAGEB6P1, COL19A1, C1R, UBXN7

Table S2: Time points, samples, key genes of triclusters and the enriched gene ontology biological processes and/ or KEGG pathways during cardiomyocytes differentiation. Functions of bold gene symbols have been described in Tables S6-S8.

Tricluster	GOBP/ KEGG Pathway	Time Points	Samples	Key Genes
41	BP: GO:0007229: integrin-mediated signaling pathway (ADAM33), GO:0043473: pigmentation (SZT2)	0day-10day-14day-20day	S1-S2	OR12D3, ADAM33, **SZT2**, ABCA13, ERI2, FANK1
42	BP: GO:0035050: embryonic heart tube development (LBX1), GO:0007368: determination of left/right symmetry (LBX1, PKD1L1), GO:0001947: heart looping (LBX1), GO:0003143: embryonic heart tube morphogenesis (LBX1), GO:0061371: determination of heart left/right asymmetry (LBX1), GO:0006508: proteolysis (CUL9, DPEP1)	0day-3day-7day-10day-20day-35day	S1-S2	**PKD1L1**, **KRTAP5-4**, **DPEP1**, CYTIP, **LBX1**, PRR20A/ B/ E, **CUL9**, **DNPH1**, RSPH10B/ B2
64	BP: GO:0042157: lipoprotein metabolic process (PEMT), GO:0018931: naphthalene metabolic process (CYP2F1), GO:0046498: S-adenosylhomocysteine metabolic process (PEMT), GO:0033146: regulation of estrogen receptor signaling pathway (DYX1C1)	10day-14day-20day-28day	S1-S2-S3	DYX1C1-CCPG1, YSK4, CYP2F1, **PEMT**, TMEM59L, RGS9BP, OR10G9, **IAPP**, **DYX1C1**, PDE11A
76	BP: GO:0014002: astrocyte development (GFAP), GO:0014010: Schwann cell proliferation (GFAP), GO:0009566: fertilization (TDRD9)	0day-3day-20day-28day	S1-S2-S3	LINC00330, **LAT**, TDRD9, BARHL2, **GIPC2**, HIST1H3J, FAM115C, KRT78, **GFAP**, **GPR50**
87	BP: GO:0048743: positive regulation of skeletal muscle fiber development (SHOX2), GO:0060272: embryonic skeletal joint morphogenesis (SHOX2), GO:0045844: positive regulation of striated muscle tissue development (SHOX2), GO:0003230: cardiac atrium development (SHOX2), GO:0003170: heart valve development (SHOX2), GO:0003205: cardiac chamber development (SHOX2). GO:0051147: regulation of muscle cell differentiation (SHOX2)	0day-3day-7day-10day-20day-28day-35day	S1-S2	MUC6, DPEP1, CUL9, **MYO5B**, SPACA5/ 5B, ZNF407, **SHOX2**

Table S3: Time points, samples, key genes of triclusters and the enriched gene ontology biological processes and/ or KEGG pathways since the day when first heart beat was observed. Functions of bold gene symbols have been described in Tables S6-S8.

Tricluster	GOBP/ KEGG Pathway	Time Points	Samples	Key Genes
8	BP: GO:0043966: histone H3 acetylation (TADA2A), GO:0016569: covalent chromatin modification (DNMT3A, TADA2A), GO:0043414: macromolecule methylation (DNMT3A)	14day-20day-28day-60day	S2-S3	LRRC37BP1, EPB41, DNMT3A, **NFATC4**, UVSSA, PTGDR2, SLC5A10, IGHV4-31, LRRC37B, TADA2A
9	BP: GO:0060263: regulation of respiratory burst (NOXO1), GO:0006801: superoxide metabolic process (NOXO1)	14day-60day-120day	S2-S3	FTH1P6, GRTP1, SPNS3, ZNF445, CEACAM5, RUNX2, RTBDN, HTR3D, NOXO1, ZNF41
44	BP: GO:0019722: calcium-mediated signaling (SELE), GO:0002687: positive regulation of leukocyte migration (SELE), GO:0005977: glycogen metabolic process (GYG2), GO:0005976: polysaccharide metabolic process (GYG2), GO:0044042: glucan metabolic process (GYG2), GO:0010517: regulation of phospholipase activity (SELE)	14day-20day-45day	S1-S2-S3	PART1, RGPD5, RGPD6, **CTHRC1**, RGPD8, GYG2, SHC1P1, SYT12, SELE, **CASP4**
63	BP: GO:0050850: positive regulation of calcium-mediated signaling (TRAT1), GO:0050851: antigen receptor-mediated signaling pathway (TRAT1)	14day-28day-60day-120day	S2-S3	ZFHX2, **AGBL3**, TRAT1, GABRQ, ACRV1, TTLL9, CMIP, SLC22A10
75	BP: GO:0006801: superoxide metabolic process (DUOX1, PREX1), GO:0006693: prostaglandin metabolic process (PDPN), GO:0006690: icosanoid metabolic process (PDPN), GO:0048286: lung alveolus development (PDPN), GO:0006692: prostanoid metabolic process (PDPN)	14day-28day-90day-120day	S2-S3	**ASB12**, **MTMR8**, CD200R1, **LGR6**, DUOX1, ZNF442, PDPN, PREX1, TGIF1, SLC22A9
86	BP: GO:0051145: smooth muscle cell differentiation (NFATC3), GO:0001569: patterning of blood vessels (NFATC3), GO:0055001: muscle cell development (NFATC3), GO:0006004: fucose metabolic process (FUT2); KP: KEGG:04370: VEGF signaling pathway (NFATC3)	14day-28day-35day	S1-S2-S3	FTH1P6, INTS6-AS1, **NFATC3**, **OLFML2B**, TTLL3, RPL14P5, **LRR1**, KIAA1328, **CTSS**, **FUT2**
98	BP: GO:0009445: putrescine metabolic process (AGMAT), GO:0009446: putrescine biosynthetic process (AGMAT), GO:0008295: spermidine biosynthetic process (AGMAT), GO:0008216: spermidine metabolic process (AGMAT), GO:0033146: regulation of estrogen receptor signaling pathway (DYX1C1), GO:0009309: amine biosynthetic process(AGMAT)	14day-20day-28day-45day-60day-90day-120day	S2-S3	DYX1C1-CCPG1, **NLRP11**, CETN4P, AWAT2, ANO9, AGMAT, SRGAP2B, UVSSA, DYX1C1, **AIDA**

Table S4: Time points, samples, key genes of triclusters and the enriched gene ontology biological processes and/ or KEGG pathways during all stages of hiPSC-derived cardiomyocyte differentiation. Functions of bold gene symbols have been described in Tables S6-S8.

Tricluster	GOBP/ KEGG Pathway	Time Points	Samples	Key Genes
24	BP: GO:0060047: heart contraction (TH, ADRBK1), GO:0003015: heart process (TH, ADRBK1), GO:0003007: heart morphogenesis (LBX1, TH), GO:0007507: heart development (LBX1, TH), GO:0035050: embryonic heart tube development (LBX1), GO:0001947: heart looping (LBX1), GO:0002026: regulation of the force of heart contraction (ADRBK1)	3day-10day-14day-35day-45day-60day-90day-120day	S1-S2-S3	**FOXJ3**, **PCDHA9**, **TH**, TRIM29, ANO9, LBX1, BOD1L1, TSSK3, **ADRBK1**, SPEF2
61	BP: GO:0000093: mitotic telophase (MAD1L1), GO:0000089: mitotic metaphase (MAD1L1), GO:0000090: mitotic anaphase (MAD1L1), GO:0001654: eye development (CRB1), GO:0001754: eye photoreceptor cell differentiation (CRB1)	0day-3day-7day-10day-14day-20day-28day-35day-45day	S1-S2	HERC2P9, CYTIP, **CRB1**, CCT8L1P, MAD1L1
79	BP: GO:0003279: cardiac septum development (FRS2), GO:0003205: cardiac chamber development (FRS2), GO:0003231: cardiac ventricle development (FRS2), GO:0003281: ventricular septum development (FRS2), GO:0000187: activation of MAPK activity (FRS2), GO:0043406: positive regulation of MAP kinase activity (FRS2)	7day-20day-28day-35day-60day-90day	S1-S2-S3	OR2T27, OR2T7, **FRS2**, NAA35, **EPS8L3**, **ICOS**, SPAG16
97	BP: GO:0044336: canonical Wnt receptor signaling pathway involved in negative regulation of apoptosis (CTNNB1), GO:0044334: canonical Wnt receptor signaling pathway involved in positive regulation of epithelial to mesenchymal transition (CTNNB1), GO:0060912: cardiac cell fate specification (CTNNB1), GO:0003306: Wnt receptor signaling pathway involved in heart development (CTNNB1), GO:0061316: canonical Wnt receptor signaling pathway involved in heart development (CTNNB1), GO:0003129: heart induction (CTNNB1), GO:0061311: cell surface receptor linked signaling pathway involved in heart development (CTNNB1); KP: KEGG:04530: tight junction (CTNNB1), KEGG:5412: Arrhythmogenic right ventricular cardiomyopathy (ARVC) (CTNNB1)	0day-3day-10day-14day-28day-45day-60day-120day	S2-S3	LRRC37A4P, RPLP0, ZNF709, **PRLH**, LRRC37A2, WDR89, OR11H6, **CCNB1IP1**, **CTNNB1**

Table S5: Time points, samples, key genes of triclusters and the enriched gene ontology biological processes and/ or KEGG pathways during maturation. Functions of bold gene symbols have been described in Tables S6-S8.

Tricluster	GOBP/ KEGG Pathway	Time Points	Samples	Key Genes
19	BP: GO:0048934: peripheral nervous system neuron differentiation (RUNX3), GO:0050680: negative regulation of epithelial cell proliferation (RUNX3), GO:0050678: regulation of epithelial cell proliferation (RUNX3)	35day-45day-60day-90day	S1-S2-S3	MYNN, LCN8, **GABRG1**, KRTAP5-5, RUNX3
43	GO:0042357: thiamine diphosphate metabolic process (TPK1), GO:0031958: corticosteroid receptor signaling pathway (NEDD4), GO:0046457: prostanoid biosynthetic process (CD74), GO:0048010: vascular endothelial growth factor receptor signaling pathway (NEDD4); KP: KEGG:00430: Taurine and hypotaurine metabolism (CSAD), KEGG:00900: Terpenoid backbone biosynthesis (IDI2)	45day-60day-90day	S1-S2-S3	KRTAP5-1, IZUMO4, MED12L, CSAD, **ANKRD13D**, **NEDD4**, IDI2, TPK1, ADAM18, **CD74**

Table S6: Genes that might be associated with cardiac disorders, cardiovascular development

Tricluster	Genes	Functions
15	*LUC7L*	Serine or arginine proteins are known to be associated with cardiac functions [1]. As *LUC7L* binds to Arg/Ser-rich domain, we hypothesize *LUC7L* as a potential drug target to impede cardiac disorders.
15	*GREB1*	It is known as estrogen responsive protein and can be used as a potential drug target as ERalpha has been reported to play an pivotal role in cardioprotection against cardiac injury [2].
15	*SYN1*	It is a well known regulator of neurotransmitter release. Hence, this finding is quite surprising to us. However a previous study reports that the concentration of the sympathetic nervous neurotransmitter is associated with failing human heart [3]. Thus, *SYN1* might be considered as a potential drug target to prevent cardiac failure.
24	*ADRBK1*	It is known to be a key regulator of LPAR1 signaling which is reported to be involved in mediating cardiomyocyte hypertrophy by a previous work [4].
24	*PCDHA9*	Protocadherin alpha-9 plays a key role in the development and maintenance of specific neuronal connections in the brain. Though it's quite surprising to us that *PCDHA9* has been identified as one of the hubs in a coexpression network, a previous study inferred the brain's effects on the heart for instance neurogenic heart diseases [5].
24	*FOXJ3*	A previous study reports that *FOXJ3* expression is associated with cardiac neural crest and thus it might provide insights into cardiovascular system development [6].
25	*BEST3*	It is known to form calcium-sensitive chloride channel which is known to be associated with heart [7,8].
26	*TCTEX1D4*	It is known to be an interaction partner of endoglin which is reported to be a potential drug target to prevent cardiac disorders [9]. Hence, we hypothesize targeting *TCTEX1D4* might provide a promising novel therapeutic approach for individuals with cardiac dysfunctions.
27	*EMR1*	It is known to be involved in cell-cell interactions which facilitates in understanding cell based therapies for restoring cardiomyocyte loss during cardiac diseases [10].
27	*NDUFAF6*	It participates in assembly of mitochondrial NADH, dehydrogenase of which plays a key role in cardiotoxicity [11]. Hence, *NDUFAF6* can be hypothesized as potential drug target to prevent heart dysfunctions or muscle damage.
27	*FAIM2*	It is a well-known regulator of Fas-mediated apoptosis in neurons and this pathway is reported to be involved in cardiac myxoma which may originate from sensory nerve tissue [12, 13]. So we hypothesize *FAIM2* as a potential drug target to prevent cardiac myxoma.
28	*CALML4*	It belongs to calmodulin family which may play pivotal role in cardiac function [7, 14].
41	*SZT2*	It may take part in superoxide dismutase which may be involved in regulating cardiac functions [7, 15].
42	*CUL9*	It is known to regulate subcellular localization of p53. A previous study inferred that mitochondrial p53 plays an important role in repairing mitochondrial DNA as a response to oxidative damage of cardiomyocyte [16]. Thus, targeting *CUL9* might prevent oxidative damage of cardiomyocyte.
42	*DPEP1*	It is known to transform leukotriene D4 to leukotriene E4 and thus involved in regulating activities of leukotrienes myocardial, sedative effects of which play a key role in cardiac dysfunction [17]. Hence, *DPEP1* can be used as a potential drug target to prevent cardiac abnormality associated with leukotrienes reactions.
42	*DNPH1*	It is known to generate purine that has cardioprotective effects against hypoxic stress [18]. Thus, *DNPH1* can be used as a potential drug target to impede hypoxic stress.
42	*KRTAP5-4*	It is quite surprising that keratin-associated protein 5-4 has been identified as a hub gene in coexpression network as it is known to be involved in forming resistant hair shaft. However, an association between mitral valve regurgitation and woolly hair (a hair shaft disorder) was inferred by a previous study [19]. Thus, we hypothesize *KRTAP5-4* as a potential drug target to prevent mitral valve regurgitation.
43	*ANKRD13D*	It is known to be involved in positive regulation of ligand-activated epidermal growth factor receptor that may be associated with cardiac diseases [7,20]. Thus, *ANKRD13D* can be hypothesized as a potential drug target to prevent cardiac disease.
43	*CD74*	It is inferred to play a crucial role in processing MHC class II antigen which is capable of detecting cardiac allograft rejection [7, 21]. Thus, *CD74* may provide insights into the context of allograft rejection.

Table S7: Genes that might be associated with cardiac disorders, cardiovascular development

Tricluster	Genes	Functions
44	*CTHRC1*	It is known to be involved in collagen deposition modification of which may result in deterioration of systolic function of heart patients [7, 22].
44	*CASP4*	It plays a pivotal role in endoplasmic reticulum (ER) stress induced apoptosis and thus can be hypothesized as a potential target to protect heart disease [7, 23].
63	*AGBL3*	A previous study reports that *AGBL3* plays an important role to process tubulin which has has been inferred to have a cardioprotective effect [7, 24].
64	*DYX1C1*	It was inferred to be a potential regulator of estrogen receptor stability; thus can be hypothesized to play an instrumental role in cardiovascular development. [25, 26].
64	*IAPP*	It can be hypothesized to play a crucial role in preventing cardiomegaly by inhibiting glycogen deposition. [27].
75	*MTMR8*	It is inferred to act on lipid which is an important enzyme in heart [7, 28].
75	*ASB12*	It is reported to be a mediator of ubiquitination and subsequent proteasomal degradation to play a cardioprotective role [7, 29].
75	*LGR6*	It enhances canonical Wnt signaling pathway which is one of the most important regulators of cardiomyocyte differentiation [7, 30].
76	*LAT*	Linker for activation of T-cells family member 1 (*LAT*) is known to be needed for T-cell antigen receptor. A previous study reports the presence of T-cells in coronary arteries of cardiac allografts from patients with graft arteriosclerosis (GA) [31]. Hence we hypothesize *LAT* as a potential drug target to prevent graft arteriosclerosis.
76	*GPR50*	*GPR50* known as melatonin-related receptor, was inferred to play a key role in leptin signaling which reduces the severity of cardiac disorders [32, 33]. Hence, *GPR50* can be hypothesized as a potential drug target to prevent cardiac dysfunctions.
79	*ICOS*	Inducible T-cell costimulator promotes the synthesis of interleukin-10 which is inferred to protect ischemic heart from reperfusion injury [34]. Hence we can hypothesize *ICOS* as a potential drug target for individuals with cardiac reperfusion injury.
79	*EPS8L3*	It is a well known interaction partner of ABI1 which has a key role in cardiovascular development [35].
86	*LRR1*	It is known to activate NF-kappaB by negatively regulating 4-1BB-mediated signaling cascades. A previous study reports that NF-kappaB is needed for adaptive cardiac hypertrophy [36]. Thus targeting *LRR1* might provide a novel insight into the treatment of cardiac hypertrophy.
86	*FUT2*	It is known to create H-antigen which is found to be pertinent to cardiac transplantation [37]. Thus, targeting *FUT2* might provide new insights into the treatment for heart transplantation.
87	*MYO5B*	It belongs to myosin family. One of the primary structural proteins of cardiac muscle is myosin, activation which plays an instrumental role in preventing systolic heart failure [38]. Hence, *MYO5B* can be hypothesized as a potential drug target to prevent cardiovascular diseases.
97	*PRLH*	Prolactin-releasing peptide is known to release prolactin (PRL) which is inferred to provide insights into prognosis of advanced chronic heart failure [39]. Hence we hypothesize *PRLH* as a potential target for patients with chronic heart failure.
97	*CCNB1IP1*	A previous study reports E3 ubiquitin-ligase family of proteins as promising targets to impede cardiac diseases [40]. As *CCNB1IP1* is a E3 ubiquitin-protein ligase, targeting this protein might provide new insights into therapeutics of heart diseases.
98	*NLRP11*	It belongs to NLRP family, member of which (NLRP3) is found to be associated with cardiac disorders [41].

Table S8: Genes that are known to be associated with cardiovascular development

Tricluster	Genes	Functions
8	*NFATC4*	A previous study infers *NFATC4* as a potential regulator in heart development [42].
15	*DHFR*	It has been identified as key gene that is known to be a key enzyme in folate mediated metabolism, playing a pivotal role in heart development [43].
15	*ZNF365*	It is known to interact with NDE1 depletion of which causes a smaller Kupffer's vesicle [44].
19	*GABRG1*	Gamma-aminobutyric acid (GABA) A receptor, gamma 1 (GABRG1) has proven effects in cardiac vagal neurons, which play a crucial role in controlling the heart rate and cardiac function in previous works [45, 46].
24	*TH*	It has been inferred in a previous study that tyrosine hydroxylase (TH) plays a key role in cardiac differentiation and primitive heart tube formation [47].
25	*TEAD1*	It is a well known regulator of proper heart development [48].
25	*PROKR2*	Prokineticin receptor-2 is reported to be associated with cardiovascular system development [49].
26	*TPH2*	It might play important roles in heart development as it generates serotonin ([5-hydroxytryptamine (5-HT)]) that is involved in regulating differentiation, proliferation during development and cardiac function [50, 51].
27	*GPER*	The role of G-protein-coupled estrogen receptor (GPER) as cardioprotective has been inferred in previous studies [52, 53].
27	*RGN*	It has been suggested in a previous study that RGN, known as regucalcin, plays an essential role in regulating heart mitochondrial function by increasing Ca2+ ATPase activity [54].
28	*WNT8A*	It is inferred to play an essential role in heart tube development [55].
28	*PPARA*	Peroxisome proliferator-activated receptor-alpha plays a critical role in heart through activating fatty acid oxidation [56].
28	*EPHB2*	Ephrin-B2 is reported to play an important role in cardiac valve maturation [57].
42	*LBX1*	It acts as a marker of embryonic stem cell induced cardiomyocyte differentiation [58].
42	*PKD1L1*	It is known to play an essential role in left/ right determination and in cardiac development [59, 60].
43	*NEDD4*	The Hect domain E3 ubiquitin ligase, *NEDD4* is known to play an instrumental role in cardiac development [61].
61	*CRB1*	It is reported to be specifically expressed in central nervous system [62].
64	*PEMT*	Inhibition of phosphatidylethanolamine N-methyltransferase (PEMT) is inferred to play an important role in preventing cardiac dysfunctions [63].
76	*GIPC2*	It is known to be an interaction partner of SEMA5A inactivation of which leads to imperfect remodeling of the cranial vascular system [64].
76	*GFAP*	Its role in cardiac valve has been established in a previous study [65].
79	*FRS2*	FRS2 phosphorylation is required for activation of MAPK pathway in cardiomyocyte development [66].
86	*NFATC3*	It plays an essential role in cardiac development [67].
86	*CTSS*	It has been suggested by a recent report that cathepsin S (CTSS) takes part in normal accumulation of autophagysomes [68]. Deficiency of autophagy has been inferred to cause heart disease [69].
86	*OLFML2B*	It is known to bind chondroitin sulfate which has been inferred to be involved in cardiac AV canal formation [70].
87	*SHOX2*	The role of SHOX2 in posterior heart field formation has been reported in a previous work [71].
97	*CTNNB1*	It is inferred to play an important role in heart development [72].
98	*AIDA*	A previous study reported that AIDA is found to be highly expressed in heart tissue [73].

Author details

References

1. Gergs U, Boknik P, Buchwalow IB, Fabritz L, Gruendker N, Kucerova D, Matus M, Werner F, Schmitz W, Neumann J: **Modulation of cardiac contractility by serine/threonine protein phosphatase type 5**. *Int J Cardiol* 2012, **154**: 116–121. doi:10.1016/j.ijcard.2010.09.009
2. Brinckmann M, Kaschina E, Altarche-Xifro W, Curato C, Timm M, Grzesiak, A, Dong J, Kappert K, Kintscher U, Unger T, Li J: **Estrogen receptor alpha supports cardiomyocytes indirectly through post-infarct cardiac c-kit+ cells**. *J Mol Cell Cardiol* 2009, **47**: 66–75. doi:10.1016/j.yjmcc.2009.03.014
3. Esler M, Lambert G, Brunner-La Rocca HP, Vaddadi G, Kaye D: **Sympathetic nerve activity and neurotransmitter release in humans: translation from pathophysiology into clinical practice**. *Acta Physiol Scand* 2003, **177**: 275–284.
4. Yang J, Nie Y, Wang F, Hou J, Cong X, Hu S, Chen X: **Reciprocal regulation of miR-23a and lysophosphatidic acid receptor signaling in cardiomyocyte hypertrophy**. *Biochim Biophys Acta* 2013, **1831**: 1386–1394. doi:10.1016/j.bbalip.2013.05.005
5. Samuels MA: **The Brainâ“Heart Connection**. *Circulation* 2007, **116**: 77–84. doi:10.1161/CIRCULATIONAHA.106.678995
6. Landgren H, Carlsson P: **FoxJ3, a novel mammalian forkhead gene expressed in neuroectoderm, neural crest, and myotome**. *Dev Dyn* 2004, **231**: 396–401.
7. Consortium TU: **Update on activities at the Universal Protein Resource (UniProt) in 2013**. *Nucleic Acids Res* 2013, **41**: 43–47. doi:10.1093/nar/gks1068
8. Zygmunt AC, Gibbons WR: **Calcium-activated chloride current in rabbit ventricular myocytes**. *Circ Res* 1991, **68**: 424–437.
9. Kapur NK, Wilson S, Yunis AA, Qiao X, Mackey E, Paruchuri V, Baker C, Aronovitz MJ, Karumanchi SA, Letarte M, Kass DA, Mendelsohn ME, Karas RH: **Reduced endoglin activity limits cardiac fibrosis and improves survival in heart failure**. *Circulation* 2012, **125**: 2728–2738. doi:10.1161/CIRCULATIONAHA.111.080002
10. Ma Z, Yang H, Liu H, Xu M, Runyan RB, Eisenberg CA, Markwald RR, Borg TK, Gao BZ: **Mesenchymal stem cell-cardiomyocyte interactions under defined contact modes on laser-patterned biochips**. *PLoS ONE* 2013, **8**: e56554. doi:10.1371/journal.pone.0056554
11. Nohl H, Gille L, Staniek K: **The exogenous NADH dehydrogenase of heart mitochondria is the key enzyme responsible for selective cardiotoxicity of anthracyclines**. *Z Naturforsch C* 1998, **53**: 279–285.
12. Terracciano LM, Mhawech P, Suess K, D'Armiento M, Lehmann FS, Jundt G, Moch H, Sauter G, Mihatsch MJ: **Calretinin as a marker for cardiac myxoma. Diagnostic and histogenetic considerations**. *Am J Clin Pathol* 2000, **114**: 754–759.
13. Liu CC, Jung SM, Orlandi A, Yeh TS, Lin YS, Shiu TF, Wu HH, Chu JJ, Lin PJ, Chu PH: **The Fas-mediated apoptotic pathway in cardiac myxoma**. *Int J Surg Pathol* 2010, **18**: 493–498.
14. Maier LS, Bers DM, Brown JH: **Calmodulin and Ca^{2+}/calmodulin kinases in the heart â“ Physiology and pathophysiology**. *Cardiovasc Res* 2007, **73**: 629–630. doi:10.1016/j.cardiores.2007.01.005
15. Kliment CR, Suliman HB, Tobolewski JM, Reynolds CM, Day BJ, Zhu X, McTiernan CF, McGaffin KR, Piantadosi CA, Oury TD: **Extracellular superoxide dismutase regulates cardiac function and fibrosis**. *J Mol Cell Cardiol* 2009, **47**: 730–742. doi:10.1016/j.yjmcc.2009.08.010
16. Nithipongvanitch R., Ittarat W, Velez JM, Zhao R, St Clair DK, Oberley TD: **Evidence for p53 as guardian of the cardiomyocyte mitochondrial genome following acute adriamycin treatment**. *J Histochem Cytochem* 2007, **55**: 629–639.
17. Burke JA, Levi R, Guo ZG, Corey EJ: **Leukotrienes C4, D4 and E4: effects on human and guinea-pig cardiac preparations in vitro**. *J Pharmacol Exp Ther* 1982, **221**: 235–241.
18. Golan O, Issan Y, Isak A, Leipziger J, Robaye B, Shainberg A: **Extracellular nucleotide derivatives protect cardiomyocytes against hypoxic stress**. *Biochem Pharmacol* 2011, **81**: 1219–1227. doi:10.1016/j.bcp.2011.02.007
19. Zandi S, Farajzadeh S: **A new cardiac manifestation associated with woolly hair: report of two cases of woolly hair, palmoplantar keratoderma, and mitral valve regurgitation**. *Int J Dermatol* 2007, **46**: 952–954.
20. Makki N, Thiel KW, Jr Miller FJ: **The epidermal growth factor receptor and its ligands in cardiovascular disease**. *Int J Mol Sci* 2013, **14**: 20597–20613. doi:10.3390/ijms141020597
21. McGhie AI, Radovancevic B, Capek P, Moore WH, Kasi L, Lamki L, Jr Clubb FJ, Frazier OH, Willerson JT: **Major histocompatibility complex class II antigen expression in rejecting cardiac allografts: detection using in vivo imaging with radiolabeled monoclonal antibody**. *Circulation* 1997, **96**: 1605–1611.
22. Lopez B, Gonzalez A, Querejeta R, Larman M, Diez J: **Alterations in the pattern of collagen deposition may contribute to the deterioration of systolic function in hypertensive patients with heart failure**. *J Am Coll Cardiol* 2006, **48**: 89–96.
23. Minamino ., Komuro I, Kitakaze M: **Endoplasmic reticulum stress as a therapeutic target in cardiovascular disease**. *Circ Res* 2010, **107**: 1071–1082. doi:10.1161/CIRCRESAHA.110.227819
24. Casini S, Tan HL, Demirayak I, Remme CA, Amin AS, Scicluna BP, Chatyan H, Ruijter JM, Bezzina CR, van Ginneken AC, Veldkamp MW: **Tubulin polymerization modifies cardiac sodium channel expression and gating**. *Cardiovasc Res* 2010, **85**: 691–700. doi:10.1093/cvr/cvp352
25. Massinen S, Tammimies K, Tapia-Paez I, Matsson H, Hokkanen ME, Soederberg O, Landegren U, Castren E, Gustafsson JA, Treuter E, Kere J: **Functional interaction of DYX1C1 with estrogen receptors suggests involvement of hormonal pathways in dyslexia**. *Hum Mol Genet* 2009, **18**: 2802–2812. doi:10.1093/hmg/ddp215
26. Du XJ: **Clues to understanding the role of estrogen receptors in mediating cardiovascular protection**. *Cardiovasc Res* 2002, **56**: 4–7.
27. Eishi Y, Takemura T, Sone R, Yamamura H, Narisawa K, Ichinohasama R, Tanaka M, Hatakeyama S:

Glycogen storage disease confined to the heart with deficient activity of cardiac phosphorylase kinase: a new type of glycogen storage disease. *Hum Pathol* 1985, **16**: 193–197.

28. Park TS, Yamashita H, Blaner WS, Goldberg IJ: **Lipids in the heart: a source of fuel and a source of toxins**. *Curr Opin Lipidol* 2007, **18**: 277–282.
29. Zolk O, Schenke C, Sarikas A: **The ubiquitin-proteasome system: focus on the heart**. *Cardiovasc Res* 2006 **70**: 410–421.
30. Lian X, Hsiao C, Wilson G, Zhu K, Hazeltine LB, Azarin SM, Raval KK, Zhang J, Kamp TJ, Palecek SP: **Robust cardiomyocyte differentiation from human pluripotent stem cells via temporal modulation of canonical Wnt signaling**. *Proc Natl Acad Sci U S A* 2012, **109**: 1848–1857. doi:10.1073/pnas.1200250109
31. Slachta CA, Jeevanandam V, Goldman B, Lin WL, Platsoucas CD: **Coronary arteries from human cardiac allografts with chronic rejection contain oligoclonal T cells: persistence of identical clonally expanded TCR transcripts from the early post-transplantation period (endomyocardial biopsies) to chronic rejection (coronary arteries)**. *J Immunol* 2000, **165**: 3469–3483.
32. Bechtold DA, Sidibe A, Saer, BR, Li J, Hand LE, Ivanova EA, Darras VM, Dam J, Jockers R, Luckman SM, Loudon AS: **A role for the melatonin-related receptor GPR50 in leptin signaling, adaptive thermogenesis, and torpor**. *Curr Biol* 2012, **22**: 70–77. doi:10.1016/j.cub.2011.11.043
33. McGaffin KR, Sun CK, Rager JJ, Romano LC, Zou B, Mathier MA, O'Doherty RM, McTiernan CF, O'Donnell CP: **Leptin signalling reduces the severity of cardiac dysfunction and remodelling after chronic ischaemic injury**. *Cardiovasc Res* 2008, **77**: 54–63.
34. Manukyan MC, Alvernaz CH, Poynter JA, Wang Y, Brewster BD, Weil BR, Abarbanell, AM, Herrmann JL, Crowe BJ, Keck AC, Meldrum DR: **Interleukin-10 protects the ischemic heart from reperfusion injury via the STAT3 pathway**. *Surgery* 2011, **150**: 231–239. doi:10.1016/j.surg.2011.05.017
35. Ring C, Ginsberg MH, Haling J, Pendergast AM: **Abl-interactor-1 (Abi1) has a role in cardiovascular and placental development and is a binding partner of the α4 integrin**. *Proc Natl Acad Sci U S A* 2011, **108**: 149–154. doi:10.1073/pnas.1012316108
36. Zelarayan L, Renger A, Noack C, Zafiriou MP, Gehrke C, van der Nagel R, Dietz R, de Windt L, Bergmann MW: **NF-kappaB activation is required for adaptive cardiac hypertrophy**. *Cardiovasc Res* 2009, **84**: 416–424. doi:10.1093/cvr/cvp237
37. Thorpe SJ, Hunt B, Yacoub M: **Expression of ABH blood group antigens in human heart tissue and its relevance to cardiac transplantation**. *Transplantation* 1991, **51**: 1290–1295.
38. Malik FI, Hartman JJ, Elias KA, Morgan BP, Rodriguez H., Brejc K, Anderson RL, Sueoka SH, Lee KH, Finer JT, Sakowicz R, Baliga R, Cox DR, Garard M, Godinez G, Kawas R, Kraynack E, Lenzi D, Lu PP, Muci A, Niu C, Qian X, Pierce DW, Pokrovskii M, Suehiro I, Sylvester S, Tochimoto T, Valdez C, Wang W, Katori T, Kass DA, Shen YT, Vatner SF, Morgans DJ: **Cardiac myosin activation: a potential therapeutic approach for systolic heart failure**. *Science* 2011, **331**: 1439–1443. doi:10.1126/science.1200113
39. Parissis JT, Farmakis D, Fountoulaki K, Rigas A, Nikolaou M, Paraskevaidis IA, Bistola V, Venetsanou K, Ikonomidis I, Anastasiou-Nana M, Kremastinos DT, Filippatos G: **Clinical and neurohormonal correlates and prognostic value of serum prolactin levels in patients with chronic heart failure**. *Eur J Heart Fail* 2013, **15**: 1122–1130. doi:10.1093/eurjhf/hft070
40. Willis MS, Schisler JC, Patterson C: **Appetite for destruction: E3 ubiquitin-ligase protection in cardiac disease**. *Future Cardiol* 2008, **4**: 65–75. doi:10.2217/14796678.4.1.65
41. Hermansson C, Lundqvist A, Wasslavik C, Palmqvist L, Jeppsson A, Hulten LM: **Reduced expression of NLRP3 and MEFV in human ischemic heart tissue**. *Biochem Biophys Res Commun* 2013, **430**: 425–428. doi:10.1016/j.bbrc.2012.11.070
42. Bushdid PB, Osinska H, Waclaw RR, Molkentin JD, Yutzey KE: **NFATc3 and NFATc4 are required for cardiac development and mitochondrial function**. *Circ Res* 2003, **92**: 1305–1313.
43. Sun S, Gui Y, Jiang Q, Song H: **Dihydrofolate reductase is required for the development of heart and outflow tract in zebrafish**. *Acta Biochim Biophys Sin (Shanghai)* 2011, **43**: 957–969. doi:10.1093/abbs/gmr098
44. Kim S, Zaghloul NA, Bubenshchikova E, Oh EC, Rankin S, Katsanis N, Obara T, Tsiokas L: **Nde1-mediated inhibition of ciliogenesis affects cell cycle re-entry**. *Nat Cell Biol* 2011, **13**: 351–360. doi:10.1038/ncb2183
45. DiMicco JA, Gale K, Hamilton B, Gillis RA: **GABA receptor control of parasympathetic outflow to heart: characterization and brainstem localization**. *Science* 1979, **204**: 1106–1109.
46. Wang J, Irnaten M, Neff RA, Venkatesan P, Evans C, Loewy AD, Mettenleiter TC, Mendelowitz D: **Synaptic and neurotransmitter activation of cardiac vagal neurons in the nucleus ambiguus**. *Ann N Y Acad Sci* 2001, **940**: 237–246.
47. Lopez-Sanchez C, BÃ¡rtulos O, MartÃnez-Campos E, Ganan C, Valenciano AI, Garcia-MartÃnez V, De-Pablo F, Hernandez-Sanchez C: **Tyrosine hydroxylase is expressed during early heart development and is required for cardiac chamber formation**. *Cardiovasc Res* 2010, **88**: 111–120. doi:10.1093/cvr/cvq179
48. Tsika RW, Ma L, Kehat I, Schramm C, Simmer G, Morgan B, Fine DM, Hanft LM, McDonald KS, Molkentin JD, Krenz M, Yang S, Ji J: **TEAD-1 overexpression in the mouse heart promotes an age-dependent heart dysfunction**. *J Biol Chem* 2010, **285**: 13721–13735. doi:10.1074/jbc.M109.063057
49. Attramadal H: **Prokineticins and the heart: diverging actions elicited by signalling through prokineticin receptor-1 or -2**. *Cardiovasc Res* 2009, **81**: 3–4. doi:10.1093/cvr/cvn306
50. Lesch KP, Araragi N, Waider J, van den Hove D, Gutknecht, L.: **Targeting brain serotonin synthesis: insights into neurodevelopmental disorders with long-term outcomes related to negative emotionality, aggression and antisocial behaviour**. *Philos Trans R Soc Lond B Biol Sci* 2012, **367**: 2426–2443. doi:10.1098/rstb.2012.0039
51. Nebigil CG, Maroteaux L: **A novel role for serotonin in heart**. *Trends Cardiovasc Med* 2001, **11**: 329–335.
52. Liu H, Pedram A, Kim JK: **Oestrogen prevents cardiomyocyte apoptosis by suppressing p38α-mediated activation of p53 and by down-regulating p53 inhibition on p38β**. *Cardiovasc Res* 2011, **89**: 119–128. doi:10.1093/cvr/cvq265
53. Deschamps AM, Murphy E: **Activation of a novel estrogen receptor, GPER, is cardioprotective in male and female rats**. *Am J Physiol Heart Circ Physiol* 2009, **297**: 1806–1813. doi:10.1152/ajpheart.00283.2009

54. Akhter T, Sawada N, Yamaguchi M: **Regucalcin increases Ca2+-ATPase activity in the heart mitochondria of normal and regucalcin transgenic rats.** *Int J Mol Med* 2006, **18**: 171–176.
55. Kwon C, Arnold J, Hsiao EC, Taketo MM, Conklin BR, Srivastava D: **Canonical Wnt signaling is a positive regulator of mammalian cardiac progenitors**. *Proc Natl Acad Sci U S A* 2007, **104**: 10894–10899.
56. Francis GA, Annicotte JS, Auwerx J: **PPAR-alpha effects on the heart and other vascular tissues**. *Am J Physiol Heart Circ Physiol* 2003, **285**: 1–9.
57. Cowan CA, Yokoyama N, Saxena A, Chumley MJ, Silvany RE, Baker LA, Srivastava D, Henkemeyer M: **Ephrin-B2 reverse signaling is required for axon pathfinding and cardiac valve formation but not early vascular development**. *Dev Biol* 2004, **271**: 263–271.
58. Schmitteckert S, Ziegler C, Kartes L, Rolletschek A: **Transcription Factor Lbx1 Expression in Mouse Embryonic Stem Cell-Derived Phenotypes**. *Stem Cells Int* 2011, **2011**: 130970. doi:10.4061/2011/130970
59. Field S, Riley KL, Grimes DT, Hilton H, Simon M, Powles-Glover N, Siggers P, Bogani D, Greenfield A, Norris DP: **Pkd1l1 establishes left-right asymmetry and physically interacts with Pkd2**. *Development* 2011, **138**: 1131–1142. doi:10.1242/dev.058149
60. Chen CM, Norris D, Bhattacharya S: **Transcriptional Control of Leftâ“Right Patterning in Cardiac Development**. *Pediatr Cardiol* 2010, **31**: 371–377. doi:10.1007/s00246-009-9610-3
61. Fouladkou, F, Lu C, Jiang C, Zhou L, She Y, Walls JR, Kawabe H., Brose N, Henkelman RM, Huang A, Bruneau BG, Rotin D: **The ubiquitin ligase Nedd4-1 is required for heart development and is a suppressor of thrombospondin-1**. *J Biol Chem* 2010, **285**: 6770–6780. doi:10.1074/jbc.M109.082347
62. den Hollander AI, Ghiani M, de Kok YJ, Wijnholds J, Ballabio A, Cremers FP, Broccoli V: **Isolation of Crb1, a mouse homologue of Drosophila crumbs, and analysis of its expression pattern in eye and brain**. *Mech Dev* 2002, **110**: 203–207.
63. Cole LK, Dolinsky VW, Dyck JR, Vance DE: **Impaired phosphatidylcholine biosynthesis reduces atherosclerosis and prevents lipotoxic cardiac dysfunction in ApoE-/- Mice**. *Circ Res* 2011, **108**: 686–694. doi:10.1161/CIRCRESAHA.110.238691
64. Fiore R, Rahim B, Christoffels VM, Moorman AF, Pueschel AW: **Inactivation of the Sema5a gene results in embryonic lethality and defective remodeling of the cranial vascular system**. *Mol Cell Biol* 2005, **25**: 2310–2319.
65. Hainfellner JA, Voigtlaender T, Stroebel T, Mazal PR, Maddalena AS, Aguzzi A, Budka H: **Fibroblasts can express glial fibrillary acidic protein (GFAP) in vivo**. *J Neuropathol Exp Neurol* 2001, **60**: 449–461.
66. Dell'Era P, Ronca R, Coco L, Nicoli S, Metra M, Presta M: **Fibroblast growth factor receptor-1 is essential for in vitro cardiomyocyte development**. *Circ Res* 2003, **93**: 414–420.
67. Bushdid PB, Osinska H, Waclaw RR, Molkentin JD, Yutzey KE: **NFATc3 and NFATc4 are required for cardiac development and mitochondrial function**. *Circ Res* 2003, **92**: 1305–1313.
68. Pan L, Li Y, Jia L, Qin Y, Qi G, Cheng J, Qi Y, Li H, Du J: **Cathepsin S deficiency results in abnormal accumulation of autophagosomes in macrophages and enhances Ang II-induced cardiac inflammation**. *PLos One* 2012, **7**: e35315. doi:10.1371/journal.pone.0035315
69. Gustafsson AB, Gottlieb RA: **Autophagy in ischemic heart disease**. *Circ Res* 2009, **104**: 150–158. doi:10.1161/CIRCRESAHA.108.187427
70. Peal DS, Burns CG, Macrae CA, Milan D: **Chondroitin sulfate expression is required for cardiac atrioventricular canal formation**. *Dev Dyn* 2009, **238**: 3103–3110. doi:10.1002/dvdy.22154
71. Blaschke RJ, Hahurij ND, Kuijper S, Just S, Wisse LJ, Deissler K, Maxelon T, Anastassiadis K, Spitzer J, Hardt SE, SchÃ¶ler H, Feitsma H, Rottbauer W, Blum M, Meijlink F, Rappold G, Gittenberger-de Groot AC: **Targeted mutation reveals essential functions of the homeodomain transcription factor Shox2 in sinoatrial and pacemaking development**. *Circulation* 2007, **115**: 1830–1838.
72. Norden J, Kispert A: **Wnt/Ctnnb1 signaling and the mesenchymal precursor pools of the heart**. *Trends Cardiovasc Med* 2012, **22**: 118–122. doi:10.1016/j.tcm.2012.07.006
73. Rui Y, Xu Z, Xiong B, Cao Y, Lin S, Zhang M, Chan SC, Luo W, Han Y, Lu Z, Ye Z, Zhou HM, Han J, Meng A, Lin SC: **A beta-catenin-independent dorsalization pathway activated by Axin/JNK signaling and antagonized by Aida**. *Dev Cell* 2007, **13**: 268–282.
